# Graphene-integrated microtube whispering-gallery mode resonators for polarization-sensitive optical modulation and photodetection

**DOI:** 10.1038/s41377-025-02097-1

**Published:** 2026-02-28

**Authors:** Tianjun Cai, Ziyu Zhang, Binmin Wu, Jiayang You, Zhi Zheng, Yunqi Wang, Changlu Bian, Yang Wang, Yuan Tian, Yuhang Chi, Qingyu Xiao, Mingze Ma, Li Chen, Junhan Liu, Xiang-zhong Chen, Enming Song, Jizhai Cui, Gaoshan Huang, Yongfeng Mei

**Affiliations:** 1 International Institute for Intelligent Nanorobots and Nanosystems & State Key Laboratory of Surface Physics, College of Intelligent Robotics and Advanced Manufacturing, Fudan University, Shanghai, China; 2 Yiwu Research Institute of Fudan University, Yiwu, Zhejiang China; 3 State Key Laboratory of Infrared Physics, Shanghai Institute of Technical Physics, Chinese Academy of Sciences, Shanghai, China; 4 Shanghai Frontiers Science Research Base of Intelligent Optoelectronics and Perception, Institute of Optoelectronics, Fudan University, Shanghai, China

**Keywords:** Optical materials and structures, Electronics, photonics and device physics

## Abstract

The monolithic photonic-electronic integration is crucial for high-bandwidth optical communication and computing, while existing structures struggle to reconcile compact footprints with performance preservation. Here, graphene-integrated silicon nitride microtube whispering-gallery mode resonators, fabricated via wafer-level nanomembrane self-rolling process, are demonstrated for polarization optical modulation and photodetection in photonic-electronic synergy. The engineered lobe-shaped architecture in the microtube facilitates axial mode quantization, greatly enhancing the optical mode confinement and improving the quality factor. A balanced trade-off between photodetection efficiency and optical resonance is achieved by adjusting the coupling between graphene and microtube resonance, and graphene-integrated microtube resonators with lobe structure demonstrate an efficient optical resonance ($Q$ = 2008.36) and high photoresponsivity (2.80 A W^−1^). Furthermore, fourfold rotational symmetry breaking in microtubes presents a workable structural paradigm for the polarization-sensitive optical modulation and photodetection, overall characteristics presents a promising platform for optical manipulation and multidimensional detection of integrated photonic and optoelectronic systems.

## Abstract (web summary)

We engineered and fabricated a graphene-integrated microtube resonator using a self-rolling nanomembrane process, achieving enhanced optical confinement and polarization-sensitive optoelectrical functionality for next-generation photonic systems.

## Graphical abstract

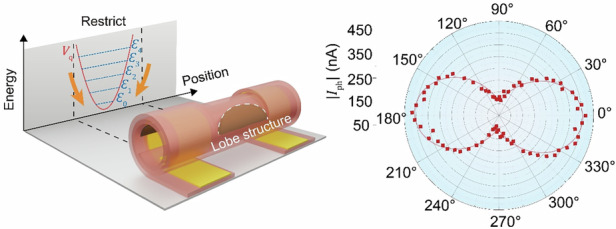

## Introduction

**Fig. 1:**
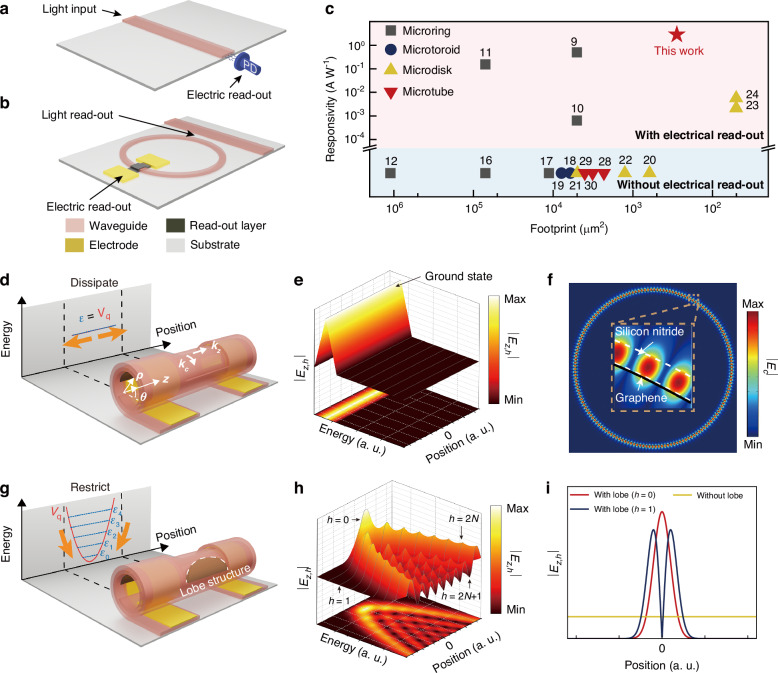
Design methodology of Gr-integrated microtube resonators. **a** The scheme of electrical read-out from box waveguide. **b** The scheme of electrical read-out from microring WGM resonator. **c** A comparison of the photoresponsivities and footprint between our work and other WGM resonators with electrical read-out integration and the footprint of some WGM resonators without electrical read-out integration. **d** The scheme of Gr-integrated microtube WGM resonator. **e** Calculation of the axial optical field distribution along the axial direction of the microtube resonator without lobe structure. **f** Simulation of optical field distribution along the circular direction (Inset: enlarged view of circular optical field distribution in the wall of microtube resonator.). **g** The scheme of Gr-integrated microtube WGM resonator with lobe structure. **h** Calculation of the axial optical field distribution along the axial direction of the microtube resonator with lobe structure. **i** Calculation of the axial optical field distribution along the axial direction of the microtube resonator with lobe structure (*h* = 0 and *h* = 1) and without lobe structure

Photonic devices demonstrate substantial advantages over electronic circuits in bandwidth, energy efficiency, and operational speed^1^. These inherent benefits make photonics ideal for high-speed signal transmission, while electronics excel at sophisticated information processing^2^. The synergistic photonic-electronic interaction supports both high-bandwidth transmission and efficient information processing^3–5^. In conventional on-chip integrated optical systems, planar dielectric waveguides coupled with photodetectors achieve optical-to-electrical signal transduction^6–8^, as illustrated in Fig. 1a. To enhance optoelectrical transduction efficiency and achieve wavelength selectivity, whispering-gallery mode (WGM) resonators are incorporated for their high-Q resonances and wavelength-specific coupling (Fig. 1b)^9–12^. Optical signals propagating through the waveguide undergo evanescent coupling into the microring resonator, where phase-matched modes enable wavelength-specific channel demultiplexing^13^ and subsequent optoelectrical conversion^14^. However, fundamental constraints of telecommunication wavelengths require microring radii of hundreds of micrometers to maintain phase-matching conditions and minimize bending losses^15^, leading to large footprints that restrict high-density integration^16,17^. Although three-dimensional WGM architectures, such as microtoroids^18,19^ and microdisks^20–24^, offer reduced footprints through vertical optical mode confinement, their fabrication complexity still faces challenges^25^, particularly in suspended structures that complicate electrical integration (Fig. 1c, Supplementary Table 1).

Low flexural rigidity of nanomembranes enables significant out-of-plane deformation into microtubes within acceptable strain ranges while retaining their intrinsic electrical and optical properties^26,27^. As a result, the strain-induced self-rolling nanomembrane offers a promising solution for photonic-electronic synergy in three-dimensional microtube resonators with a small footprint. Leveraging this strain-engineering capability, various on-chip applications have been successfully demonstrated, including three-dimensional microtube WGM resonators^28–30^ and polarization-sensitive photodetectors^26,31^. Silicon nitride (SiN_x_) satisfies the fundamental requirements for efficient light modulation in optical cavities, including low propagation losses^32,33^ and broad transparency windows^34^, while offering CMOS compatibility^35,36^ and precisely controllable residual strain^37,38^ for strain-engineered self-rolling applications^39^. Meanwhile, to realize fully photonic-electronic functional devices, integrating efficient photodetection elements is essential, requiring materials that can effectively convert optical signals into electrical outputs while maintaining compatibility with the self-rolling process. The atomic-scale thickness of graphene (Gr)^40^ provides minimal optical absorption, ensuring negligible cavity perturbation^8,41^, exceptional carrier mobility^42^, and high optoelectrical conversion efficiency^43,44^. Meanwhile, graphene can sustain strains up to 25% before mechanical failure^45^, while significant modifications to its electronic properties typically require strains exceeding 10%^46^. Previous demonstrations have explored graphene in self-rolled microtubes for various applications: graphene-based photodetectors with gate modulation^47^, molecular sensing platforms^48^, and photoresponse enhancement in SiGe rolled-up microtubes^27^. Furthermore, the chemical stability, environmental durability^49^, and mechanical flexibility of Gr make it an ideal platform for the integration in self-rolling photonic-electronic systems^27,47^.

In this work, we demonstrate a photonic-electronic platform based on Gr-integrated SiN_x_ microtube WGM resonators fabricated via a strain-induced self-rolling process. Based on Born-Oppenheimer approximation and simulation via finite element methods, we investigate the optical field distribution within the resonant cavity. Guided by these optical field distribution analyses, we incorporate an engineered lobe structure to introduce discrete energy levels in the microtube and enhance axial optical field confinement, resulting in a quality factor significantly higher than conventional microtube resonators. The precise control over microtube radius is achieved through engineering the thickness of the nanomembrane, enabling a tunable resonant spectrum. Gr is integrated into the microtube resonators to enable electrical read-out of the confined optical signals. With an adjustable integration length of Gr, the balance between optical resonance performance and photoresponse efficiency can be optimized to suit various photonic-electronic application scenarios. Furthermore, the fourfold asymmetry of the microtube structure induces polarization sensitivity in the Gr-integrated microtube resonator. The polarization-dependency can be theoretically investigated through an anisotropic model of the two-dimensional Gr crystalline structure in conjunction with a statistical electric field approximation model and validated through experimental characterization of polarization-sensitive optical resonance and photoresponse. This integrated platform presents a sophisticated solution for next-generation photonic circuits demanding precise optical manipulation and efficient optoelectrical conversion with polarization sensitivity.

## Results

In the design of photonic-electronic synergy resonators, we begin with theoretical calculations and numerical simulations of Gr-integrated SiN_x_ microtube resonator structure to analyze the optical field distribution within the microtube resonator. As illustrated in Fig. 1d, optical signals coupling into the microtube resonator induce partial absorption by Gr^50,51^, converted to an output photocurrent signal. The fabrication of the device leverages pre-strained SiN_x_ with pre-transferred Gr to construct a dual-terminal photoresponse self-rolling microtube structure, integrating the Gr layer within the walls of the microtube resonators (Fig. S1).

In a simplified two-dimensional model of WGM resonators, selective coupling occurs when the wavelength ${{\rm{\lambda }}}_{m}$ satisfies the resonance condition given by Eq. 1:

1 $${\lambda }_{m}=\frac{2\pi R{n}_{\mathrm{eff}}}{m},m\in {N}^{* }$$

where *R* is the radius of the WGM microring resonator, ${n}_{\mathrm{eff}}$ represents its effective refractive index, and $m$ is the azimuthal mode number. Compared to planar microring resonators, the optical field axial propagation in three-dimensional microtube resonators is non-negligible, relying on the introduction of a three-dimensional model. Consequently, the wave vector $k$ within the microtube resonator is decomposed into the axial direction ${k}_{z}$ and the circular direction ${k}_{\mathrm{c}}$. The Born-Oppenheimer approximation^52^ can be introduced to separate the axial (${E}_{z}\left(z\right)$) and circular (${E}_{\mathrm{c}}\left(\rho ,\theta ,z\right)$) components of the optical field $E\left(\rho ,\theta ,z\right)$ within the microtube resonators:

2 $$E\left(\rho ,\theta ,z\right)={E}_{z}\left(z\right){E}_{\mathrm{c}}\left(\rho ,\theta ,z\right)$$

In conventional microtube resonators, the symmetry characteristic maintains constant ${E}_{z}\left(z\right)$ along the $z$-axial direction (as illustrated in Fig. 1e), resulting in unrestricted axial propagation (as illustrated in Fig. 1d). This unimpeded propagation causes significant energy dissipation, ultimately degrading the Q factor of the resonator. The circular component ${E}_{\mathrm{c}}\left(\rho ,\theta ,z\right)$ satisfies the harmonic equation (Supplementary Note 1):

3 $$-\frac{1}{{n}_{\mathrm{eff}}^{2}}{\nabla }_{\rho ,\theta }^{2}{E}_{\mathrm{c}}\left(\rho ,\theta ,z\right)={k}_{\mathrm{c}}^{2}\left(z\right){E}_{\mathrm{c}}\left(\rho ,\theta ,z\right)$$

yielding eigenvalues ${k}_{\mathrm{c},m}=m/{n}_{\mathrm{eff}}\left(z\right)R$, which is consistent with Eq. 1. Normalized eigenfunctions take the form *E*_*c,m*_=*E*_0,*m*_(*ρ*)exp[*ik*_*c,m*_(z)*n*_eff_(*z*)*Rθ*], where *E*_0,*m*_(ρ) represents the normalized amplitude (dimensionality: ${LM}{T}^{-3}{I}^{-1}$). This equation can be solved via finite element method, and the solution is a periodic electric field, consistent with the formation of standing wave (Fig. 1f). The optical field is constricted around the wall of the microtube, and part of the optical field distribution region overlaps with the Gr attached to the inner wall of the SiN_x_ microtube, ensuring that a part of the optical field can be absorbed by Gr. The axial component ${E}_{z}\left(z\right)$ obeys the quasi-Schrödinger equation (Supplementary Note 1):

4 $$\left\{\begin{array}{l}{\hat{H}}_{\mathrm{q}}{E}_{z}\left(z\right)={\mathcal{E}}{E}_{z}\left(z\right)\\ {\hat{H}}_{\mathrm{q}}=\sqrt{-{\left(\frac{\hslash c}{{n}_{\mathrm{eff}}}\frac{\partial }{\partial z}\right)}^{2}+{V}_{\mathrm{q}}^{2}}\end{array}\right.$$

where ${\hat{H}}_{\mathrm{q}}$ is the quasi-Hamiltonian, ${V}_{\mathrm{q}}\left(z\right)=\hslash c{k}_{\mathrm{c}}\left(z\right)$ is the quasi-potential, and ${\mathcal{E}}=\hslash {ck}$ is photon energy. The self-adjoint nature of operator $-{\left(\frac{\hslash c}{{n}_{\mathrm{eff}}}\frac{\partial }{\partial z}\right)}^{2}+{V}_{\mathrm{q}}^{2}$ guarantees unique solutions, with $-{\left(\frac{\hslash c}{{n}_{\mathrm{eff}}}\frac{\partial }{\partial z}\right)}^{2}$ and ${V}_{\mathrm{q}}^{2}$ being positive definite operators. ${E}_{z}$ remains dimensionless while ${E}_{\mathrm{c}}$ maintains dimensions of electrical field intensity. As for traditional WGM analysis, the axial light propagation is out of consideration and $-{\left(\frac{\hslash c}{{n}_{\mathrm{eff}}}\frac{\partial }{\partial z}\right)}^{2}$ in the quasi-Hamiltonian becomes zero. In that case, ${\hat{H}}_{q}={V}_{q}=\hslash c{k}_{c}$ and ${\mathcal{E}}=\hslash c{k}_{c}$. Then we can rewrite Eq. 3 as $-\frac{{\hslash }^{2}{c}^{2}}{{n}_{\mathrm{eff}}^{2}}{\nabla }_{\rho ,\theta }^{2}E\left(\rho ,\theta ,z\right)={{\mathcal{E}}}^{2}E\left(\rho ,\theta ,z\right)$, which is the same as traditional coupled mode analysis.

The introduction of lobe structures, which is shown in Fig. 1g, will make the geometry structure of the microtube change along the axis. The optical path traverses both air and the microtube wall, where additional self-rolling turns result in an increased effective refractive index ${n}_{\mathrm{eff}}$^53^. Essentially, as shown in Fig. S2, the pathway of light within the self-rolling microtube resonators is influenced by the self-rolling turns, which determines the effective refractive index of the resonators, so continuous variation of self-rolling turns can create a gradient of effective refractive index. Therefore, by engineering curved lobe structures, a curved effective refractive index distribution can be constructed, which manifests as a curved potential surface ${V}_{\mathrm{q}}={V}_{0}+{V}_{2}{z}^{2}$, where ${V}_{0}$ is the quasi-potential at $z=0$, corresponding to the vertex of the parabola, while ${V}_{2}$ represents its quadratic coefficient. This potential results in linearly independent eigenfunctions ${E}_{\mathrm{z},h}$ with distinct quantum numbers $h$ and corresponding eigenvalues ${{\mathcal{E}}}_{h}$ (Fig. 1h; Supplementary Note 2; Fig. S3). The energy levels ${{\mathcal{E}}}_{h}$ are discrete along the axial direction, so the axial optical field of the microtube resonator with lobe structure is restricted at the vicinity of local maximum values (as illustrated in Fig. 1i), thereby weakening the energy dissipation deriving from axial field propagation and enhancing the Q factor of the microtube resonator. Therefore, the lobe structure promotes the light transmission and modulation ability of optical field in the microtube resonators.

**Fig. 2:**
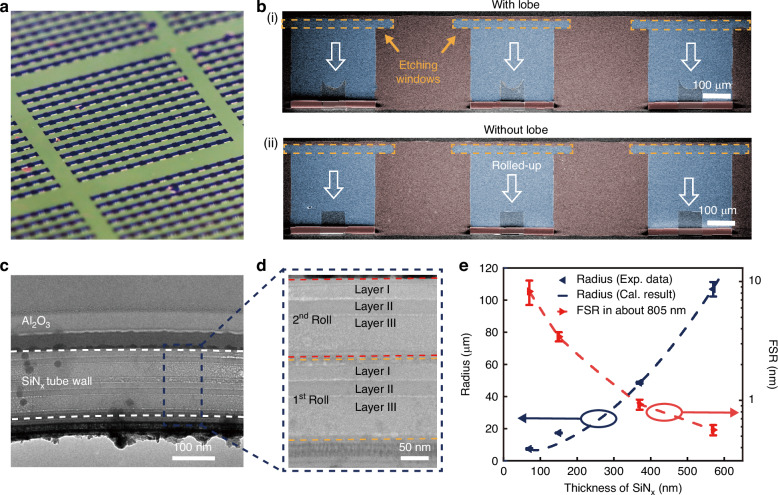
Fabrication and characterization of microtube resonators. **a** Optical image of microtube resonator arrays on a 2-inch wafer. **b** SEM image of microtube resonator arrays (i) with lobe structure and (ii) without lobe structure. **c** TEM image of the microtube wall. **d** Enlarged TEM image of multilayer structure in the microtube wall. **e** Left axis: the relationship between microtube radius and SiN_x_ thickness. Right axis: the relationship between FSR at about 805 nm and SiN_x_ thickness

Guided by the aforementioned theoretical analysis, the wafer-scale fabrication of microtube resonator arrays is achieved through strain-engineered self-rolling process (Fig. 2a, Figure S4), with a yield of 97.92% and radius variation coefficient of 2.88%. The strain gradient in SiN_x_ nanomembranes stem from the variation in radio-frequency (RF) of plasma-enhanced chemical vapor deposition (PECVD)^54^. The self-rolling mechanism originates from controlled release of vertical strain gradients in nanomembranes, where SiN_x_ nanomembranes undergo directional rolling from pre-patterned etching windows to form three-dimensional microtube architectures. Subsequently, a pre-defined etch-stop region is implemented at the terminal positions of the self-rolling trajectory, strategically maintaining the optical propagation region of microtubes in freestanding configuration (Fig. 2b). This design feature effectively prevents optical transmission induced by wall-substrate contact, thereby suppressing energy dissipation channels. Concurrent with geometric control, the parabolic patterns are engineered to induce corresponding lobe structures along microtube axes. These architectural modifications create gradient refractive index profiles that enforce axial energy quantization through discrete eigenstates, as predicted by the theoretical model. To validate structural integrity and interfacial quality, we performed cross-sectional analysis using focused ion beam (FIB) milling combined with transmission electron microscopy (TEM). Figure 2c presents a side view of the microtube via TEM, showing conformal 80 nm Al_2_O_3_ encapsulation layers deposited by atomic layer deposition (ALD). This protective coating ensures mechanical stability during FIB processing while maintaining optical confinement properties. In the higher magnification characterization (Fig. 2d), we can observe that the nanomembrane has rolled into two turns. The compact contact between layers minimizes interfacial gaps, thereby reducing optical field dissipation and ensuring low-loss light propagation within the microtube walls. Meanwhile, the penetration of XeF_2_ is an important impact to the performance of the device, so the FIB milled sample is also characterized by TEM- energy-dispersive X-ray spectroscopy EDX. As shown in Figure S5, the EDX mapping clearly demonstrates that fluorine species are predominantly confined within the Al_2_O_3_ protective layer, with minimal penetration into the SiN_x_ layers. This experimental evidence confirms that the Al_2_O_3_ coating effectively prevents XeF_2_ from defecting the rolled microtube structure while allowing selective etching of the exposed Ge sacrificial layer. The radii of WGM resonant cavities are of great significance to their performance. Therefore, a series of SiN_x_ nanomembranes with thickness variations is fabricated. Both the SiN_x_ layer I (deposited at low RF power) and layer II (deposited at middle RF power) maintain a fixed thickness of 35 nm, while layer III (deposited at high RF power) is fabricated with four distinct thicknesses, including 0 nm (sample #1), 80 nm (sample #2), 300 nm (sample #3), and 500 nm (sample #4). This configuration enables modulation of the total nanomembrane thickness from 70 nm to 570 nm. Optical microscopy analysis (Figure S6) revealed corresponding microtube radii of 7.4 ± 0.5 μm, 17.3 ± 0.3 μm, 48.6 ± 0.6 μm, and 106.8 ± 4.5 μm for the respective total thicknesses. These experimental results align with theoretical predictions from the Nikishkov multilayer self-rolling model, which can be expressed as^55^:

5 $$\left\{\begin{array}{c}c=\frac{{\sum }_{i=1}^{n}{E}_{i}{t}_{i}{\varepsilon }_{i}^{0}}{{\sum }_{i=1}^{m}{E}_{i}{t}_{i}}\\ {y}_{\mathrm{b}}=\frac{{\sum }_{i=1}^{n}{E}_{i}{t}_{i}\left({y}_{i}+{y}_{i-1}\right)}{2{\sum }_{i=1}^{n}{E}_{i}{t}_{i}}\\ R=\frac{2{\sum }_{i=1}^{n}{E}_{i}{t}_{i}\left[{y}_{i}^{2}+{y}_{i}{y}_{i-1}+{y}_{i-1}^{2}-3{y}_{\mathrm{b}}\left({y}_{i}+{y}_{i-1}-{y}_{\mathrm{b}}\right)\right]}{3{\sum }_{i=1}^{m}{E}_{i}{t}_{i}\left({y}_{i}+{y}_{i-1}-2{y}_{\mathrm{b}}\right)\left(c-{\varepsilon }_{i}^{0}\right)}\end{array}\right.$$

where $i$ is the index of layer, ${t}_{i}$ is the thickness of the $i$-th layer of the nanomembrane, and ${y}_{i}$ is the height of the $i$-th layer relative to the horizontal plane, satisfying the equations ${y}_{0}=0$ nm and ${y}_{i}={y}_{i-1}+{t}_{i}$. ${{\rm{\varepsilon }}}_{i}^{0}$ is the initial strain of the nanomembrane before self-rolling, $n$ is the total number of layers in the multilayer strained nanomembrane, and ${E}_{i}$ is the Young’s modulus of the $i$-th layer. In our study, the Young’s moduli of the three layers of SiN_x_ nanomembranes can be considered as the same, allowing Eq. 5 to be simplified to:

6 $$R=\frac{{\left({t}_{1}+{t}_{2}+{t}_{3}\right)}^{3}}{6\left[{t}_{1}{t}_{2}\Delta {\varepsilon }_{12}+{t}_{1}{t}_{3}\left(\Delta {\varepsilon }_{12+}\Delta {\varepsilon }_{23}\right)+{t}_{2}{t}_{3}\Delta {\varepsilon }_{23}\right]}$$

with $\Delta {{\rm{\varepsilon }}}_{12}={{\rm{\varepsilon }}}_{1}-{{\rm{\varepsilon }}}_{2}$ and $\Delta {{\rm{\varepsilon }}}_{23}={{\rm{\varepsilon }}}_{2}-{{\rm{\varepsilon }}}_{3}$ denoting interlayer strain gradients. The radii of the microtubes depend on the thickness of each layer as well as the strain differences between adjacent layers. Based on Eq. 6, we fitted the experimental data to obtain $\Delta {{\rm{\varepsilon }}}_{12}=0.58 \%$ and $\Delta {{\rm{\varepsilon }}}_{23}=$0.51%. As shown in the left axis of Fig. 2e, the calculated predictions are consistent with the experimental results. Based on the strain analysis and radius prediction model, we can manipulate the radius of microtubes via modulating the thickness of SiN_x_ nanomembranes. The photoluminescence (PL) spectra of these samples are measured and analyzed using Lorentzian deconvolution, as illustrated in Fig. S7. As shown in the right axis of Fig. 2e, the free spectra range (FSR) in about 805 nm of these four samples are measured, which are 8.25 ± 1.92 nm (sample #1), 3.43 ± 0.31 nm (sample #2), 0.92 ± 0.09 nm (sample #3), and 0.56 ± 0.06 nm (sample #4), respectively. The FSRs of these samples decrease as their radii increase, which means smaller mode volumes and stronger optical field confinement capabilities, and it is consistent with Eq. 1. Above all, the resonant wavelengths of the microtube resonators are tunable via designing the thickness of self-rolling nanomembranes. Considering the increasing thickness of nanomembranes will lead to higher fabrication complexity and lower FSR that makes the resonant peaks harder to recognize, sample #1 with the total thickness of 70 nm will be chosen for the following discussion.

**Fig. 3:**
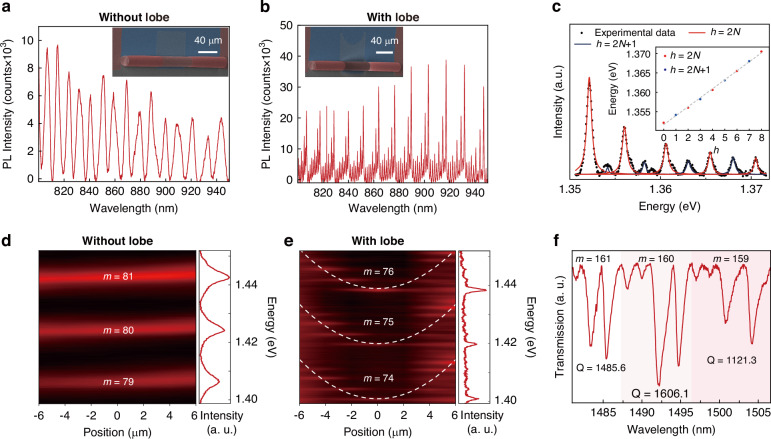
Optical resonant modulation characteristics of microtube resonators. **a** The PL spectrum of microtube resonators without lobe structure. (Inset: the SEM image of a microtube resonator without lobe structure.) **b** The PL spectrum of microtube resonators with lobe structure (Inset: the SEM image of a microtube resonator with lobe structure.). **c** The axial modes of microtube resonator with lobe structure when circular $m=79$ (Inset: linear fitting of the axial energy levels.). **d** Left: The linear PL spectra scanning along the axial direction of the microtube resonator without lobe structure. Right: PL spectrum when focusing on the middle of the microtube without lobe structure. **e** Left: The linear PL spectra scanning along the axial direction of the microtube resonator with lobe structure. Right: PL spectrum when focusing on the middle of the microtube with lobe structure. **f** The transmission spectrum of the fiber taper coupled with the microtube resonator with lobe structure

Enhanced confinement of the resonators facilitates the formation of standing waves for optical signals that satisfy the resonant conditions, thereby promoting efficient coupling with the microtube resonator. This confinement capability of the WGM resonators can be quantitatively characterized by the Q factor. To evaluate the resonator performance, PL measurements were conducted to determine the Q factor of the WGM microtube resonators. As described in Eq. 1, when the incident light wavelength matches the resonant conditions, standing waves are established within the resonator, resulting in significantly enhanced light-matter interactions. This enhancement manifests as distinct resonant peaks in the PL spectrum. The Q factor of resonant peaks can be quantified by:

7 $$Q=\frac{{\lambda }_{\mathrm{P}}}{\Delta {\lambda }_{\mathrm{P}}}$$

where ${{\rm{\lambda }}}_{\mathrm{P}}$ is the center wavelength of the resonant peak and $\Delta {{\rm{\lambda }}}_{\mathrm{P}}$ is its full width at half maximum (FWHM). It is worth noting that the self-rolling microtube structures are more accurately described as microrolls or microscrolls. However, our self-rolled structures typically complete multiple turns (Fig. 2d), creating an effectively closed optical path where light propagating along the circumferential direction encounters a quasi-continuous circular waveguide, enabling the formation of standing wave patterns characteristic of WGM resonators, which is also verified by FEM simulation of a double-turns self-rolling structure (as shown in Fig. S8), where a periodic electric field is formatted along the circumferential direction. Figure 3a shows the PL spectrum of the microtube resonator without a lobe structure with the Q factor of about 300. In these structures, every circular resonant mode m only has one resonant peak due to its single state at the axial field distribution. The PL spectrum of the microtube resonator with lobe structure is shown in Fig. 3b. The maximum Q factor of resonators can reach up to 3191.21. In the PL spectrum, every peak of circular resonant mode is split into several peaks due to the discrete energy levels ${{\mathcal{E}}}_{h}^{m}$ along the axial direction. Figure 3c shows the resonant peaks with the circular resonant mode $m=79$. Theoretically, there is no odd energy level distributed in the middle of the microtube (Fig. S9a). Meanwhile, the energy density of the incident light exhibits a Gaussian distribution from the center point outward, so part of it overlaps with the region corresponding to odd energy levels (Fig. S9b), and odd energy levels are also observed. The linear relationship between axial energy levels and their mode numbers is observed, consistent with equidistant energy levels shown in Supplementary Note 1. It is worth noting that the lobe structure is designed at the end of a self-rolling pathway for process simplicity, but the position of the lobe structures does not influence the phenomenon of discrete axial energy levels. As shown in Fig. S10, the lobe structure is designed at the middle of the microtube wall, and the discrete axial energy levels are also observed in its PL spectra. To further observe the axial energy states, the PL line scanning along the axis of the microtube is conducted. Figure 3d shows that the axial energy states of the microtube resonator without a lobe structure only include a ground state, and the optical field is free to propagate along the axial direction. In contrast, discrete axial energy levels are observed in Fig. 3e, which restricts the light propagation along the axial direction and decreases its energy dissipation, resulting in higher Q factor. These experimental results are consistent with the calculation results shown in Fig. 1f, h, confirming the discrete energy levels deriving from the lobe structure and the restriction effect on the axial optical field propagation. Furtherly, the finite-difference time-domain (FDTD) method is introduced to simulate the resonating spectrum of the microtube resonator with lobe structure. As shown in Fig. S11, the results reveal discrete energy levels along the axial direction, showing excellent agreement with the experimental results. To verify the light coupling ability of microtube resonators, a fiber taper is used to approach the microtube resonator (Figure S12 shows experimental structure). The transmission spectrum is shown in Fig. 3f. The axial discrete energy states are also observed in the transmission spectrum with $m$ = 161, 160, 159, and the maximum Q factor reaches 1705.45. These results demonstrate the efficient coupling capability between microtube WGM resonators and fiber tapers, indicating promising potential for fiber-based photonic applications^56,57^.

**Fig. 4:**
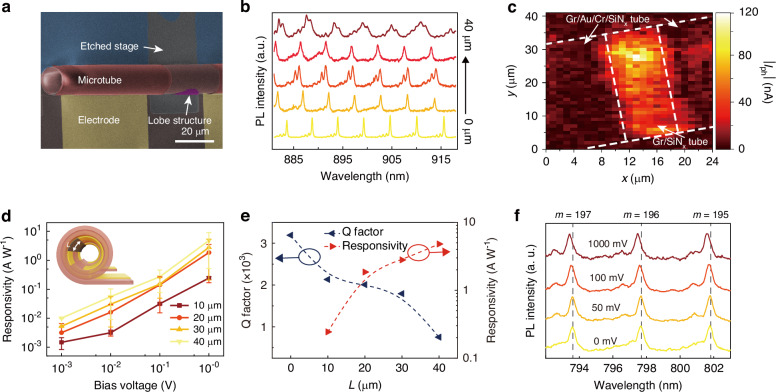
Electrical read-out and modulation characteristics of the Gr-integrated microtube resonators. **a** The SEM image of a Gr-integrated microtube resonator. **b** The PL spectrum of Gr-integrated microtube resonators with different $L$. **c** Spatially resolved photocurrent measurements of the Gr-integrated microtube WGM resonator. **d** The responsivities of Gr-integrated microtubes with different Gr-integration length $L$ under variable bias voltage. (Inset: the schematic diagram of the Gr-integration model.) **e** The Q factors (right axis) and photoresponsivities (left axis) of Gr-integrated microtube resonators with different $L$. **f** The PL spectrum of Gr-integrated microtube resonators under different bias voltages

The integration of an electrical read-out layer into optical systems makes them promising for real-time transmission^58^, processing^59^, and storage of optical signals^60,61^, while on-chip integration facilitates broader application scenarios^62^ and enhanced integration density. To achieve electrical read-out of optical signals coupled into the microtube resonator, Gr is incorporated into the channel region of the resonator structure. As shown in Fig. 4a, Cr/Au electrodes are prepared on both ends of Gr, where Gr detects the optical signals and Cr/Au electrodes subsequently read out the electrical signals. The Gr characteristics are analyzed using Raman line scanning along the microtube axis (shown in Fig. S13). The Raman spectrum exhibits distinct G and G’ peaks, which serve as characteristic spectral fingerprints of Gr. The absence of a prominent D peak indicates minimal defects within the Gr lattice structure^63^. To investigate the influence of Gr on optical resonance and optoelectrical response, a set of Gr-integrated microtube resonators is designed with varying Gr-integration lengths $L$ ranging from 0 μm to 40 μm. The PL spectra of four types of samples integrated with Gr resonance are characterized, as shown in Fig. 4b. To elucidate the underlying photoresponse mechanism, comprehensive characterizations of optoelectronic properties are conducted across both frequency and spatial domains. In the frequency domain, spectral responsivity measurements are systematically performed across the visible to near-infrared wavelength range (450-800 nm), as quantitatively depicted in Fig. S14. The microtube exhibited optoelectrical conversion efficiency throughout the investigated spectral window. In the spatial domain, spatially resolved photocurrent mapping is implemented to determine the precise photoactive regions (Fig. 4c). The spatially resolved measurements reveal that photocurrent generation is predominantly localized within the Gr channel region, where optical standing wave resonances are established. The photoresponsivities of four types of samples integrated with Gr are characterized under bias voltages varying from 1 mV to 1 V. As shown in Fig. 4d, the responsivities are linearly correlated to the bias voltage, demonstrating that the photoresponse is primarily governed by photoconductive mechanisms. The responsivity characterization under the bias voltage of 1 V reveals values of 0.24 A W^−^^1^, 1.85 A W^−^^1^, 2.80 A W^−1^ and 4.80 A W^−1^ for the respective samples (as illustrated in the right axis of Fig. 4e). The results indicate that photoresponse decreases as $L$ decreases, and the photoresponse is weak when $L$ is down to 10 μm. It is worth noting that the external quantum efficiency is larger than 100% when $L >$ 20 μm. The photoconductive gain mechanism inherently enables EQE that exceeds the theoretical limit of photovoltaic devices and an enhanced responsivity, as each absorbed photon can trigger the circulation of multiple charge carriers through the external circuit before recombination occurs. Considering the balance between photoresponse and Q factor, the subsequent experiments will be conducted in the sample with $L$ set as 30 μm. The Q factors of the PL spectra shown in Fig. 4b are calculated through Lorentzian fitting. The maximum Q factors of the 5 samples mentioned above are 3191.21, 2134.28, 2008.36, 1792.64, and 752.91, as illustrated in the left axis of Fig. 4e. As $L$ increases, the Q factor of the microtube resonators decreases, and the intensity of resonant peaks declines when $L$ increases to 40 μm. Notably, two sharp decreases in Q factor occurred during the increase of $L$. The first decrease occurred between $L$ = 0 μm and $L$ = 10 μm, attributed to the introduction of Gr, which absorbed the optical field propagating in the microtube wall and impacted the confinement capability of the lobe structure against axial field dissipation (Supplementary Note 3), as evidenced by the PL line scanning shown in Fig. S15. The second decrease occurs between $L$ = 30 μm and $L$ = 40 μm. When $L$ exceeds 40 μm, Gr extends into the second winding and further into the central area with dense optical field, where the higher optical field intensity leads to enhanced absorption and greater energy loss in the resonator. To verify the capability of the device to operate in telecommunication applications, the noise current and detectivity of the device is also measured. As shown in Fig. S16a, the noise measurement is conducted across the frequency range from 1 Hz to ${10}^{5}$ Hz, revealing predominantly 1/$f$ noise characteristics with noise current levels ranging from 10^−^^22^ to 10^−27 ^A² Hz^−1^, indicating relatively low noise performance. As shown in Fig. S16b, the detectivity (${D}^{* }=\frac{R}{\sqrt{\frac{4{k}_{b}T}{{R}^{{\prime} }A}+\frac{2e{I}_{{dark}}}{A}}})$ at the wavelength range from 450 nm to 800 nm is also calculated with ${I}_{{dark}}=$ 9.95 × 10^−5^A and the highest detectivity of 1.99 × 10^7^ Jones. Meanwhile, as shown in Figure S17, the photoresponse dynamics is measured at four representative wavelengths: 520 nm, 638 nm, 940 nm and 1550 nm, demonstrating that our SiN_x_ microtube photodetectors exhibit response times on the order of ~100 μs across visible and telecommunication wavelengths.

In addition to optoelectronic coupling, a constant voltage can also be applied to the electrodes to modulate the PL peak, introducing an additional degree of freedom for optical selection of microtube resonators. To characterize the electro-optical modulation capability, the PL spectrum of the Gr-integrated microtube resonator with different bias voltage applied on Gr is characterized, including 0 mV, 50 mV, 100 mV, 1000 mV (shown in Fig. 4f). The resonating peaks ${{\rm{\lambda }}}_{\mathrm{P}}$ deviate after the bias voltage is applied. We calculate the peak deviations under different bias voltages (shown in Fig. S18). The bias voltage leads to the blue shift of the peaks and this phenomenon is more obvious at higher bias voltage^64,65^. We conduct linear fitting to the relationship between $\frac{1}{{\lambda }_{\mathrm{P}}}$ and $m$ (shown in Fig. S19) and calculate the slopes ${k}_{\mathrm{slope}}$ of the linear fitting curves. Results show that ${k}_{\mathrm{slope}}$ increases from 6.4225 mm^−1^ to 6.4310 mm^−1^ when the bias voltage increases from 0 mV to 1000 mV. Combining with Eq. 1, the relationship between ${k}_{\mathrm{slope}}$ and ${n}_{\mathrm{eff}}$ is:

8 $${n}_{\mathrm{eff}}=\frac{1}{2\pi R{k}_{\mathrm{slope}}}$$

which indicates the decrease of ${n}_{\mathrm{eff}}$ as the bias voltage increases, which is consistent with the negative thermos-optic coefficient of Gr^66^. Combining with the excellent electrical conductivity of Gr and the low heat capacity of its self-rolling structure, this structure can also be regarded as an ideal carrier for electro-optical modulation photonic devices. Furthermore, our work demonstrates a proof-of-concept three-dimensional microtube resonator platform where the specific waveguide material can be strategically selected based on application requirements. The self-rolling methodology and lobe design are not limited to SiN_x_ and can accommodate various photonic materials with superior electro-optic properties. For instance, silicon-based microtubes would enable carrier density modulation through gate voltage control, providing enhanced modulation efficiency leveraging mature silicon photonics processes^4^. Alternatively, ferroelectric materials such as lithium niobate offer intrinsically strong electro-optic coefficients, enabling efficient voltage-controlled refractive index modulation^21,22^. The versatility of our nanomembrane self-rolling approach thus provides a flexible platform for integrating diverse materials optimized for specific performance metrics, addressing both Q factor and modulation requirements through material engineering rather than structural limitations.

**Fig. 5:**
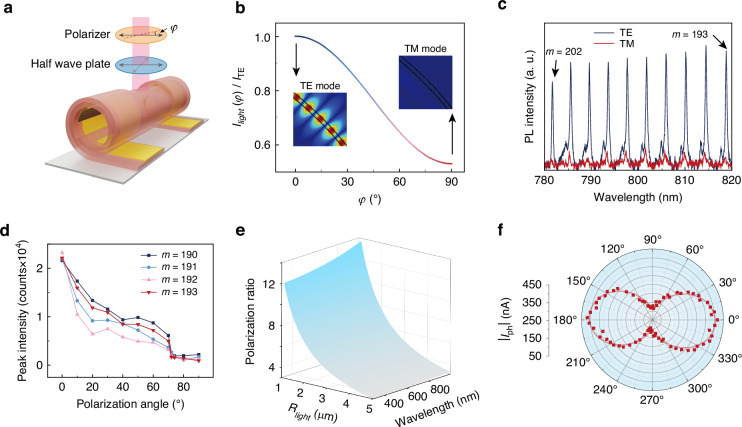
The polarization characteristics of Gr-integrated microtube resonators. **a** The scheme of polarization response testing experiments for Gr-integrated microtube resonators. **b** Polarization angular dependence of average light intensity within the wall of microtube. (Inset: simulation of TE mode and TM mode optical field distribution in the microtube.) **c** The TE and TM mode PL spectrum of microtube resonator. **d** The resonant peak intensities of PL spectrum with different polarization angles. **e** Calculated polarization ratio dependence on radius of light spot and wavelength of incident light. **f** The photoresponse of Gr-integrated microtube resonator with incident light at different polarization angles

Geometrically, the planar structure of the nanomembrane exhibits fourfold rotational symmetry in the in-plane direction, which becomes broken during the self-rolling process, leading to distinct interactions between the self-rolling microtube and different polarized light, which is material-agnostic and depends primarily on the geometric asymmetry of the nanomembrane rather than intrinsic material properties. To investigate the polarization sensitivity of the Gr-integrated microtube resonator, we employ linearly polarized light as the incident source (Fig. 5a, Fig. S20a). The polarization angle $\varphi$ is defined as the angle between the electric field intensity vector and the microtube axis. Specifically, the average light intensity within the microtube walls for transverse electric (TE) mode (${I}_{\mathrm{TE}}$) and transverse magnetic (TM) mode (${I}_{\mathrm{TM}}$) can be described by our previous work^27,31,67^:

9 $$\left\{\begin{array}{c}{I}_{\mathrm{TE}}=\frac{1}{2}{\varepsilon }_{0}c{E}_{\mathrm{ex}}^{2}\\\,\,\,\,\,\,\, {I}_{\mathrm{TM}}=\frac{1}{2}{\varepsilon }_{0}{E}_{\mathrm{ex}}^{2}\frac{1+{\varepsilon }_{\mathrm{r}}^{2}}{2{\varepsilon }_{\mathrm{r}}^{2}}\end{array}\right.$$

where ${E}_{\mathrm{ex}}$ is the external electric field intensity and ${\varepsilon }_{r}$ is the relative permittivity of SiN_x_. Therefore, according to the principle of superposition of electric fields, the relationship between $\varphi$ and the average light intensity (${I}_{\mathrm{light}}\left(\varphi \right)$) can be calculated (as illustrated in Fig. 5b):

10 $${I}_{\mathrm{light}}\left(\varphi \right)=\frac{1}{2}{\varepsilon }_{0}c{\left({E}_{\mathrm{ex}}\cos \varphi \right)}^{2}+\frac{1}{2}{\varepsilon }_{0}{\left({E}_{\mathrm{ex}}\sin \varphi \right)}^{2}\frac{1+{\varepsilon }_{\mathrm{r}}^{2}}{2{\varepsilon }_{\mathrm{r}}^{2}}={I}_{\mathrm{TE}}\left(1+\frac{1-{\varepsilon }_{\mathrm{r}}^{2}}{2{\varepsilon }_{\mathrm{r}}^{2}}{\sin }^{2}\varphi \right)$$

To further investigate the polarization-sensitive resonance of microtube resonators, the electric field distribution of TE mode and TM mode is simulated via finite element method. For TE mode, the electric field intensity is parallel to the axis of the microtube, in which the electric field can be sustained in the resonator wall and forms the standing wave. In contrast, for TM mode, the electric field intensity is perpendicular to the axis of the microtube, in which the thickness of the resonator wall is much smaller than the wavelength. Therefore, the electric field cannot be sustained in the resonator wall and the standing wave cannot be formed. PL spectra of the Gr-integrated microtube resonators with different polarization angles $\varphi$, varying from ${0}^{\circ }$ to ${90}^{\circ }$, are characterized to verify the theoretical results (Fig. S20b). Figure 5c shows the PL spectrum of TE mode ($\varphi ={0}^{\circ }$) and TM mode ($\varphi ={90}^{\circ }$) incident light. The resonate peak intensities of TE mode are much higher than those of TM mode, and the peak intensities decrease as the polarization angle $\varphi$ increases from ${0}^{\circ }$ to ${90}^{\circ }$ (as shown in Fig. 5d), with the optical polarization ratio of ~10–20. Meanwhile, due to the higher in-plane absorption coefficient of Gr^27^, and the fact that the electrical field intensity of TE mode light is parallel to the Gr surface, there is enhanced interaction between TE mode light and Gr. In contrast, the electric field intensity direction of TM mode light is perpendicular to the Gr, which means the Gr is almost opaque to the light. A complex permittivity model (Fig. S21) is conducted to study the angular dependence of device absorptivity (Supplementary Note 4):

11 $$\left\{\begin{array}{l}\alpha \left(\beta \right)=\frac{2\pi c}{\lambda }\sqrt{\sqrt{{\left({\varepsilon }_{\mathrm{Si}{\mathrm{N}}_{\mathrm{x}}}-A{\cos }^{2}\beta \right)}^{2}+{\left(\lambda \frac{{\sigma }_{\mathrm{Si}{\mathrm{N}}_{\mathrm{x}}}+2A\varGamma {\cos }^{2}\beta }{2\pi c}\right)}^{2}}-\left({\varepsilon }_{\mathrm{Si}{\mathrm{N}}_{\mathrm{x}}}-A{\cos }^{2}\beta \right)}\\ A=\frac{{\mu }_{\mathrm{c}}{e}^{2}}{\pi {\hslash }^{2}\left({\omega }^{2}+4{\varGamma }^{2}\right){\varepsilon }_{0}\Delta } > 0\end{array}\right.$$

where $\beta$ is the angle between the electrical field intensity and the Gr surface, ${\varepsilon }_{\mathrm{Si}{\mathrm{N}}_{\mathrm{x}}}$ and ${\sigma }_{\mathrm{Si}{\mathrm{N}}_{\mathrm{x}}}$ are the permittivity and conductivity of SiN_x_, respectively, ${\mu }_{\mathrm{c}}$ is the chemical potential of the material, $e$ is the electron charge, $\omega$ is the angular frequency of the photon, $\hslash$ is the reduced Planck constant, $\varGamma$ is the scattering rate and $\Delta$ is the thickness of the nanomembrane. As illustrated in Fig. S22, the angular dependence of device absorptivity $\alpha \left(\beta \right)$ reveals distinct polarization-dependent characteristics. Maximum absorption occurs when the electric field intensity $\widetilde{E}$ is parallel to the surface of Gr (*α* = 0°, 90° and 360°), while minimum absorption is observed when $\widetilde{E}$ is perpendicular to the surface of Gr ($\alpha =\,$90° and 270°). Based on these results, the polarization ratio can be calculated (see Supplementary Note 5):

12 $$\left\{\begin{array}{l}\mathrm{ratio}\left(\lambda \right)=\alpha \left(0\right)\frac{{\int }_{-\frac{l}{2}}^{\frac{l}{2}}{\int }_{0}^{\frac{\pi }{2}}{I}_{\mathrm{in}}\left(\rho ,\,\beta ,\,z\right){\rm{d}}\beta {\rm{d}}z}{{\int }_{-\frac{l}{2}}^{\frac{l}{2}}{\int }_{0}^{\frac{\pi }{2}}\alpha \left(\beta \right){I}_{\mathrm{in}}\left(\rho ,\,\beta ,\,z\right){\rm{d}}\beta {\rm{d}}z}\\ {I}_{\mathrm{in}}\left(\rho ,\beta ,z\right)=\left\{\begin{array}{l}1,\,{\left(\rho \cos \beta -R\right)}^{2}+{z}^{2}\le {r}_{\mathrm{in}}\\ 0,\,{\left(\rho \cos \beta -R\right)}^{2}+{z}^{2} > {r}_{\mathrm{in}}\end{array}\right.\end{array}\right.$$

where ${I}_{\mathrm{in}}\left(\rho ,\beta ,z\right)$ represents the spatial intensity distribution of the incident light, ${r}_{\mathrm{in}}$ is its beam radius, and $l$ is the length of the photoresponse channel. As illustrated in Fig. S23, the polarization ratio exhibits wavelength dependence arising from the dispersive permittivity of SiN_x_^68^, maintaining an average value of approximately 4.30 across the visible and infrared spectral range. The polarization ratio also relies on ${r}_{\mathrm{in}}$ (as illustrated in Fig. 5e). As ${r}_{\mathrm{in}}$ decreases, the incident light focuses more on the region where $\beta$ is near to 90°and exhibits higher polarization ratio, and the polarization ratio can be more than 13 when ${r}_{\mathrm{in}}$ lowers to 1 μm, where the incident light is basically focused on the position parallel to the Gr. The polarization-resolved photoresponse of the Gr-integrated microtube resonator is characterized by using a polarized 520 nm laser source with ${r}_{\mathrm{in}}\approx$ 3 μm (Fig. 5f). The experimentally determined polarization ratio of 4.25 shows good agreement with the theoretical prediction of 4.27. It is worth noting that both the resonant characteristics and photoresponse of Gr-integrated microtube WGM resonators exhibit significant polarization sensitivity, highlighting their potential applications in polarization-selective coupling and detection schemes as well as in wavelength division multiplexing systems.

## Discussion

In conclusion, we have designed and fabricated wafer-scale Gr-integrated SiN_x_ microtube WGM resonators using nanomembrane self-rolling. The engineered lobe structure introduces discrete axial energy levels that effectively constrain optical field propagation, substantially enhancing resonator performance. Our theoretical framework based on the Born-Oppenheimer approximation, establishes a new paradigm for understanding three-dimensional optical confinement in self-rolled architectures. The tunable balance between photodetection efficiency and optical resonance performance, combined with intrinsic polarization sensitivity from structural asymmetry, provides versatile functionality for photonic-electronic applications.

The nanomembrane self-rolling platform represents a versatile technological foundation with broad application potential. Beyond photonic integration, this approach has proven successful in photodetectors^27,31,47^, MEMS systems^69^, radio-frequency transformers^38^, ferroelectric memory^70^, and nanorobots^71^, as summarized in Supplementary Table 2. Future developments could incorporate alternative two-dimensional materials for enhanced spectral responsivity, multiple resonator arrays for wavelength-division multiplexing, and quantum photonic applications exploiting polarization encoding capabilities.

Our platform establishes multiple pathways for next-generation photonic-electronic systems. The structural symmetry-breaking mechanism provides a universal design principle applicable to various photonic architectures. Near-term applications include on-chip optical sensing, neuromorphic computing, and polarimetric detection systems. The compatibility with standard CMOS processes enables industrial scalability, addressing needs for cost-effective photonic device manufacturing. This work provides a scalable foundation for realizing complex three-dimensional integrated photonic systems with unprecedented functionality and miniaturization.

## Materials and Methods

### Fabrication of Gr-integrated microtube resonators

The corresponding fabrication process involves utilizing a bottom-up approach to prepare and pattern multilayer nanomembranes of Ge/Al_2_O_3_/SiN_x_/Cr/Au/Gr/Al_2_O_3_. Among these, the 20 nm Ge and 10 nm/20 nm Cr/Au nanomembranes were prepared using e-beam evaporation (DE 400) with the rate of 0.05 nm s^−^^1^ and 0.1 nm s^−1^, respectively. The SiN_x_ nanomembranes consist of three layers grown by plasma-enhanced chemical vapor deposition (PECVD, PlasmaPro System 100) at different radio frequencies. Specifically, layer I was deposited at the pressure of 12 mTorr (RF power 0 W, ICP power 20 W, SiH_4_:N_2_ = 13.5:10) and layer II was deposited at the same pressure of 12 mTorr (RF power 10 W, ICP power 20 W, SiH_4_: N_2_ = 13.5: 10). Then layer III was deposited at the pressure of 12 mTorr (RF power 20 W, ICP power 20 W, SiH_4_: N_2_ = 13.5: 10). Gr is prepared using the wet transfer method. Poly (methyl methacrylate) (PMMA) A7 was pre-spin-coated on Gr at 1500 rpm for 30 s, and then heated on a hot plate at 180 ^◦^C for 30 min. Then, the PMMA/Gr/copper foil was immersed in copper etchant (HCl: H_2_O: H_2_O_2_ = 10:20:3.75) for 10 min to release PMMA/Gr from the copper foil. The released PMMA/Gr was cleaned by deionized water rinsing 3 times and transferred onto the SiN_x_/Cr/Au patterns. The Al_2_O_3_ coating layer was deposited via ALD (H1410174) at 300 ^◦^C. By employing XeF_2_ gas to etch the Ge sacrificial layer, the strain in the SiN_x_ nanomembranes is released, causing the various layers of the nanomembranes to roll up.

### Characterization of nanomembrane and microtube resonators

Morphological characteristics of the Gr/SiN_x_ nanomembranes and microtube resonators were performed via SEM Zeiss Sigma 300. TEM characterization of the microtube’s cross-section was performed by JEOL ARM200F. Raman spectra of Gr nanomembrane were performed by Renishaw inVia. The photoelectrical properties of Gr read-out layers were performed by Keysight B2902B at room temperature. Polarization-resolved photodetection was performed by Metatest MStarter 200.

### PL spectrum experiments testing microtube resonators

The PL spectrum was conducted by the PL mode of confocal laser scanning Raman spectrometer (Renishaw inVia Qontor) with 1800 cm^−1^ slit and 532 nm laser. The optical waves capable of coupling with the resonator are those whose wavelengths satisfy the criteria for the WGM resonator. Consequently, resonance peaks appear in the PL spectrum at these corresponding wavelengths, indicating efficient coupling and energy transfer processes occurring within the resonator.

### Finite element modeling of electric field distribution

The simulation of the electrical field distribution was conducted by finite element methods via COMSOL Multiphysics. The model consisted of 70 nm SiN_x_/1 nm Gr microtube and circular perfect match layer with a thickness of 2 µm. The outside radius of the microtube is set at 8 µm and that of the circular perfect match layer is set at 15 µm.

## Supplementary information

Supplementary information for Graphene-Integrated Microtube Whispering-Gallery Mode Resonators for Polarization-Sensitive Optical Modulation and Photodetection

## Data Availability

The data that support the findings of this study are available from the corresponding author upon request.

## References

[1] Coldren, L. A., Corzine, S. W. & Mašanović, M. L. Diode Lasers and Photonic Integrated Circuits. (Hoboken: John Wiley & Sons Inc., 2012).

[2] Ning, S. P. et al. Photonic-electronic integrated circuits for high-performance computing and ai accelerators. *J. Lightwave Technol.* **42**, 7834–7859 (2024).

[3] Sun, C. et al. Single-chip microprocessor that communicates directly using light. *Nature* **528**, 534–538 (2015). 26701054 10.1038/nature16454

[4] Atabaki, A. H. et al. Integrating photonics with silicon nanoelectronics for the next generation of systems on a chip. *Nature* **556**, 349–354 (2018). 29670262 10.1038/s41586-018-0028-z

[5] Haffner, C. et al. Low-loss plasmon-assisted electro-optic modulator. *Nature* **556**, 483–486 (2018). 29695845 10.1038/s41586-018-0031-4 PMC5935232

[6] Wang, D. et al. Enhancing the graphene photocurrent using surface plasmons and a p-n junction. *Light Sci. Appl.* **9**, 126 (2020). 32704359 10.1038/s41377-020-00344-1 PMC7371713

[7] Gao, Y. et al. Graphene-on-silicon nitride waveguide photodetector with interdigital contacts. *Appl. Phys. Lett.* **112**, 211107 (2018).

[8] Guo, J. S. et al. High-performance silicon-graphene hybrid plasmonic waveguide photodetectors beyond 1.55 μm. *Light Sci. Appl.* **9**, 29 (2020). 32140220 10.1038/s41377-020-0263-6 PMC7048841

[9] Schuler, S. et al. High-responsivity graphene photodetectors integrated on silicon microring resonators. *Nat. Commun.* **12**, 3733 (2021). 34145226 10.1038/s41467-021-23436-x PMC8213857

[10] Wu, J. H. et al. Dual-function optical modulation and detection in microring resonators integrated graphene/MoTe_2_ heterojunction. *Appl. Phys. Rev.* **11**, 021426 (2024).

[11] Zhang, Q. et al. High-responsivity MoS_2_ hot-electron telecom-band photodetector integrated with microring resonator. *Appl. Phys. Lett.* **120**, 261111 (2022).

[12] Zhang, Y. N. et al. Design and optimization of four-wave mixing in microring resonators integrated with 2D graphene oxide films. *J. Lightwave Technol.* **39**, 6553–6562 (2021).

[13] Liu, D. J. et al. Silicon photonic filters. *Microw. Optical Technol. Lett.* **63**, 2252–2268 (2021).

[14] Chang, K. L. et al. Graphene-integrated waveguides: properties, preparation, and applications. *Nano Res.* **15**, 9704–9726 (2022).

[15] Kavokin, A. V. et al. Microcavities. 2nd edn. (Oxford: Oxford University Press, 2017).

[16] Bai, B. W. et al. Microcomb-based integrated photonic processing unit. *Nat. Commun.* **14**, 66 (2023). 36604409 10.1038/s41467-022-35506-9 PMC9814295

[17] Lu, J. J. et al. Ultralow-threshold thin-film lithium niobate optical parametric oscillator. *Optica* **8**, 539–544 (2021).

[18] Gao, H. W. et al. 3D printed on-chip microtoroid resonators and nested spiral photonic devices. *Photonics Res.* **9**, 1803–1810 (2021).

[19] Jiang, X. F. et al. Chaos-assisted broadband momentum transformation in optical microresonators. *Science* **358**, 344–347 (2017). 29051375 10.1126/science.aao0763

[20] Kippenberg, T. J. et al. Demonstration of an erbium-doped microdisk laser on a silicon chip. *Phys. Rev. A* **74**, 051802 (2006).

[21] Wang, C. et al. Integrated high quality factor lithium niobate microdisk resonators. *Opt. Express* **22**, 30924–30933 (2014). 25607041 10.1364/OE.22.030924

[22] Wang, J. et al. High-*Q* lithium niobate microdisk resonators on a chip for efficient electro-optic modulation. *Opt. Express* **23**, 23072–23078 (2015). 26368411 10.1364/OE.23.023072

[23] Wang, T. J. et al. High-quality LiNbO_3_ microdisk resonators by undercut etching and surface tension reshaping. *Opt. Express* **20**, 28119–28124 (2012). 23263047 10.1364/OE.20.028119

[24] Xu, X. J. et al. Microdisk enhanced photodetector based on Ge self-assembled quantum dots on silicon-on-insulator. *Thin Solid Films* **557**, 363–367 (2014).

[25] Yang, S. C., Wang, Y. & Sun, H. D. Advances and prospects for whispering gallery mode microcavities. *Adv. Optical Mater.* **3**, 1136–1162 (2015).

[26] Zhang, Z. Y. et al. Multilevel design and construction in nanomembrane rolling for three-dimensional angle-sensitive photodetection. *Nat. Commun.* **15**, 3066 (2024). 38594254 10.1038/s41467-024-47405-2 PMC11004118

[27] Zhang, Z. Y. et al. Graphene readout silicon-based microtube photodetectors for encrypted visible light communication. *Adv. Mater.* **37**, 2413771 (2025). 10.1002/adma.202413771 39573846

[28] Kong, Y. et al. Integration of a metal-organic framework film with a tubular whispering-gallery-mode microcavity for effective CO_2_ sensing. *ACS Appl. Mater. Interfaces* **13**, 58104–58113 (2021). 34809420 10.1021/acsami.1c16322

[29] Madani, A. et al. Optical microtube cavities monolithically integrated on photonic chips for optofluidic sensing. *Opt. Lett.* **42**, 486–489 (2017). 28146508 10.1364/OL.42.000486

[30] Yang, S. et al. Enhanced evanescent field coupling of smart particles in tubular optical microcavity for sensing application. *Adv. Optical Mater.* **10**, 2102158 (2022).

[31] Wu, B. M. et al. One-step rolling fabrication of VO_2_ tubular bolometers with polarization-sensitive and omnidirectional detection. *Sci. Adv.* **9**, eadi7805 (2023). 37851806 10.1126/sciadv.adi7805 PMC10584336

[32] Xiang, C., Jin, W. & Bowers, J. E. Silicon nitride passive and active photonic integrated circuits: trends and prospects. *Photonics Res.* **10**, A82–A96 (2022).

[33] Mao, S. C. et al. Low propagation loss SiN optical waveguide prepared by optimal low-hydrogen module. *Opt. Express* **16**, 20809–20816 (2008). 19065219 10.1364/oe.16.020809

[34] Blumenthal, D. J. et al. Silicon nitride in silicon photonics. *Proc. IEEE* **106**, 2209–2231 (2018).

[35] Saggau, C. N. et al. Wafer-scale high-quality microtubular devices fabricated via dry-etching for optical and microelectronic applications. *Adv. Mater.* **32**, 2003252 (2020). 10.1002/adma.202003252 32686201

[36] Liu, J. Q. et al. High-yield, wafer-scale fabrication of ultralow-loss, dispersion-engineered silicon nitride photonic circuits. *Nat. Commun.* **12**, 2236 (2021). 33863901 10.1038/s41467-021-21973-z PMC8052462

[37] Huang, J. Y. et al. Enhanced photothermoelectric conversion in self-rolled tellurium photodetector with geometry-induced energy localization. *Light Sci. Appl.* **13**, 153 (2024). 38965220 10.1038/s41377-024-01496-0 PMC11224300

[38] Huang, W. et al. Three-dimensional radio-frequency transformers based on a self-rolled-up membrane platform. *Nat. Electron.* **1**, 305–313 (2018).

[39] Huang, J. Y. et al. Nanomembrane-assembled nanophotonics and optoelectronics: from materials to applications. *J. Phys.: Condens. Matter* **35**, 093001 (2023). 10.1088/1361-648X/acabf3 36560918

[40] Wu, B. M. et al. Progress and challenges on 3D tubular structures and devices of 2D materials. *Appl. Phys. Lett.* **121**, 060503 (2022).

[41] Liu, M. et al. A graphene-based broadband optical modulator. *Nature* **474**, 64–67 (2011). 21552277 10.1038/nature10067

[42] Novoselov, K. S. et al. Electric field effect in atomically thin carbon films. *Science* **306**, 666–669 (2004). 15499015 10.1126/science.1102896

[43] Grigorenko, A. N., Polini, M. & Novoselov, K. S. Graphene plasmonics. *Nat. Photonics* **6**, 749–758 (2012).

[44] Wang, F. et al. Gate-variable optical transitions in graphene. *Science* **320**, 206–209 (2008). 18339901 10.1126/science.1152793

[45] Lee, C. et al. Measurement of the elastic properties and intrinsic strength of monolayer graphene. *Science* **321**, 385–388 (2008). 18635798 10.1126/science.1157996

[46] Pereira, V. M., Castro Neto, A. H. & Peres, N. M. R. Tight-binding approach to uniaxial strain in graphene. *Phys. Rev. B* **80**, 045401 (2009).

[47] Deng, T. et al. Three-dimensional graphene field-effect transistors as high-performance photodetectors. *Nano Lett.* **19**, 1494–1503 (2019). 30698978 10.1021/acs.nanolett.8b04099

[48] Ma, Z. et al. Self-rolling of monolayer graphene for ultrasensitive molecular sensing. *ACS Appl. Mater. Interfaces* **13**, 49146–49152 (2021). 34617726 10.1021/acsami.1c12592

[49] Zheng, Y. K. et al. Graphene strain-effect transistor with colossal on/off current ratio enabled by reversible nanocrack formation in metal electrodes on piezoelectric substrates. *Nano Lett.* **23**, 2536–2543 (2023). 36996350 10.1021/acs.nanolett.2c04519

[50] Gabor, N. M. et al. Hot carrier-assisted intrinsic photoresponse in graphene. *Science* **334**, 648–652 (2011). 21979935 10.1126/science.1211384

[51] Song, J. C. W. et al. Hot carrier transport and photocurrent response in graphene. *Nano Lett.* **11**, 4688–4692 (2011). 21936568 10.1021/nl202318u

[52] Strelow, C. et al. Light confinement and mode splitting in rolled-up semiconductor microtube bottle resonators. *Phys. Rev. B* **85**, 155329 (2012).

[53] Strelow, C. et al. Three dimensionally confined optical modes in quantum-well microtube ring resonators. *Phys. Rev. B* **76**, 045303 (2007).

[54] Huang, W. et al. On-chip inductors with self-rolled-up SiN_*x*_ nanomembrane tubes: a novel design platform for extreme miniaturization. *Nano Lett.* **12**, 6283–6288 (2012). 23171136 10.1021/nl303395d

[55] Nikishkov, G. P. Curvature estimation for multilayer hinged structures with initial strains. *J. Appl. Phys.* **94**, 5333–5336 (2003).

[56] Yu, H. Y. et al. All-optical image transportation through a multimode fibre using a miniaturized diffractive neural network on the distal facet. *Nat. Photonics* **19**, 486–493 (2025).

[57] Gao, Z. et al. Optical semantic communication through multimode fiber: from symbol transmission to sentiment analysis. *Light Sci. Appl.* **14**, 60 (2025). 39848979 10.1038/s41377-024-01726-5 PMC11758025

[58] Shekhar, S. et al. Roadmapping the next generation of silicon photonics. *Nat. Commun.* **15**, 751 (2024). 38272873 10.1038/s41467-024-44750-0 PMC10811194

[59] Chen, M. J. et al. I/O-efficient iterative matrix inversion with photonic integrated circuits. *Nat. Commun.* **15**, 5926 (2024). 39009562 10.1038/s41467-024-50302-3 PMC11251023

[60] Zhang, Z. X. et al. Advances in machine-learning enhanced nanosensors: from cloud artificial intelligence toward future edge computing at chip level. *Small Struct.* **5**, 2300325 (2024).

[61] Pan, G. Z. et al. Harnessing the capabilities of VCSELs: unlocking the potential for advanced integrated photonic devices and systems. *Light Sci. Appl.* **13**, 229 (2024). 39227573 10.1038/s41377-024-01561-8 PMC11372081

[62] Li, R. J. et al. Photonics for neuromorphic computing: fundamentals, devices, and opportunities. *Adv. Mater.* **37**, 2312825 (2025). 10.1002/adma.202312825 39011981

[63] Ferrari, A. C. & Basko, D. M. Raman spectroscopy as a versatile tool for studying the properties of graphene. *Nat. Nanotechnol.* **8**, 235–246 (2013). 23552117 10.1038/nnano.2013.46

[64] Hu, J. K. et al. 2D graphene oxide: a versatile thermo-optic material. *Adv. Funct. Mater.* **34**, 2406799 (2024).

[65] Li, J. Y. et al. High-performance graphene-integrated thermo-optic switch: design and experimental validation. *Optical Mater. Express* **10**, 387–396 (2020).

[66] Agrawal, A. Thermo-optic coefficient of chemical vapor deposited graphene multilayers. *Vidyodaya J. Sci.* **26**, 01 (2023).

[67] Wu, B. M. et al. Self-rolled-up ultrathin single-crystalline silicon nanomembranes for on-chip tubular polarization photodetectors. *Adv. Mater.* **35**, 2306715 (2023). 10.1002/adma.202306715 37721970

[68] Beliaev, L. Y. et al. Optical, structural and composition properties of silicon nitride films deposited by reactive radio-frequency sputtering, low pressure and plasma-enhanced chemical vapor deposition. *Thin Solid Films* **763**, 139568 (2022).

[69] Khandelwal, A. et al. Self-rolled-up aluminum nitride-based 3D architectures enabled by record-high differential stress. *ACS Appl. Mater. Interfaces* **14**, 29014–29024 (2022). 35700345 10.1021/acsami.2c06637

[70] Guo, Y. T. et al. Self-rolling-up enabled ultrahigh-density information storage in freestanding single-crystalline ferroic oxide films. *Adv. Funct. Mater.* **33**, 2213668 (2023).

[71] Wu, Y. et al. Nanomembrane on graphene: delamination dynamics and 3D construction. *ACS Nano* **19**, 331–344 (2025). 39748669 10.1021/acsnano.4c07589

