# Supplementary material for: Graphene-integrated microtube whispering-gallery mode resonators for polarization-sensitive optical modulation and photodetection
Source: Light Sci Appl. 2026 Feb 28;15:130. doi: 10.1038/s41377-025-02097-1 (PMC12949986; doi:10.1038/s41377-025-02097-1)
Supplement: Supplementary file 1 — Supplementary information for Graphene-Integrated Microtube Whispering-Gallery Mode Resonators for Polarization-Sensitive Optical Modulation and Photodetection [file 41377_2025_2097_MOESM1_ESM.docx]

Supporting Information for

Graphene-Integrated Microtube Whispering-Gallery Mode Resonators for Polarization-Sensitive Optical Modulation and Photodetection

Tianjun Cai^#^, Ziyu Zhang^#^, Binmin Wu^∗^, Jiayang You, Zhi Zheng, Yunqi Wang, Changlu Bian, Yang Wang, Yuan Tian, Yuhang Chi, Qingyu Xiao, Mingze Ma, Li Chen, Junhan Liu, Xiang-zhong Chen, Enming Song, Jizhai Cui, Gaoshan Huang, Yongfeng Mei^∗^

^#^ These authors contributed equally

^*^ **Email:** yfm@fudan.edu.cn, wubinmin@mail.sitp.ac.cn.

Supplementary Figures & Tables


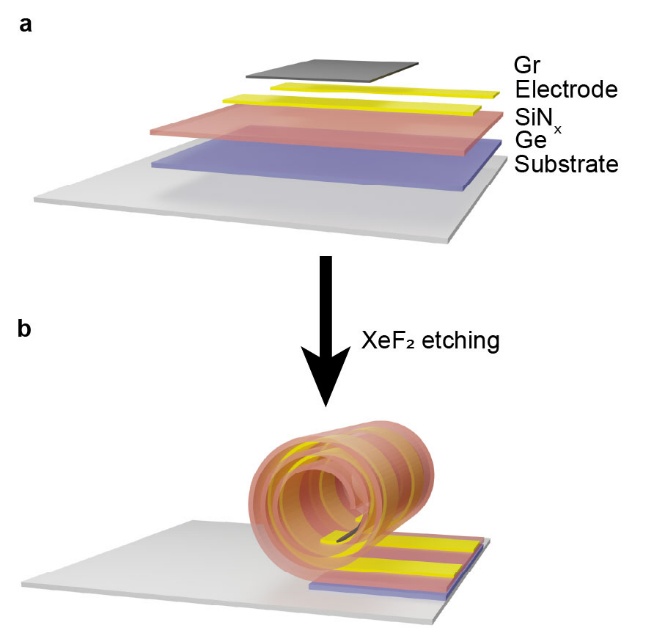


**Figure S1**. Self-rolling process of microtubes. **a** The multi-layer structure of the nanomembrane system. Ge layer is deposited by e-beam evaporation and works as the sacrificial layer. SiN_x_ layers consist of three layers deposited by plasma enhanced chemical vapor deposition with different radio frequencies and work as the waveguide. The electrode consists of a Cr layer and an Au layer also deposited by e-beam evaporation. Gr is transferred by wet transfer method. **b** Following XeF_2_ etching, the Ge layer is etched gradually, and the strain gradient result from different radio frequencies can lead to the self-rolling of multilayer SiN_x_.


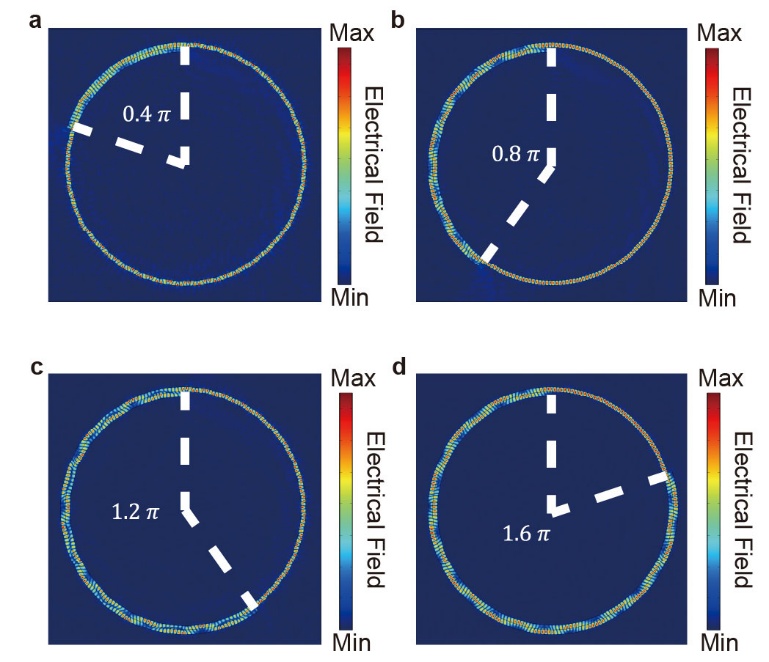


**Figure S2.** The simulated electrical field distribution in the self-rolling microtube with different overlap angle of **a** $0.4\pi$ **b** $0.8\pi$ **c** $1.2\pi$ **d** $1.6\pi$.


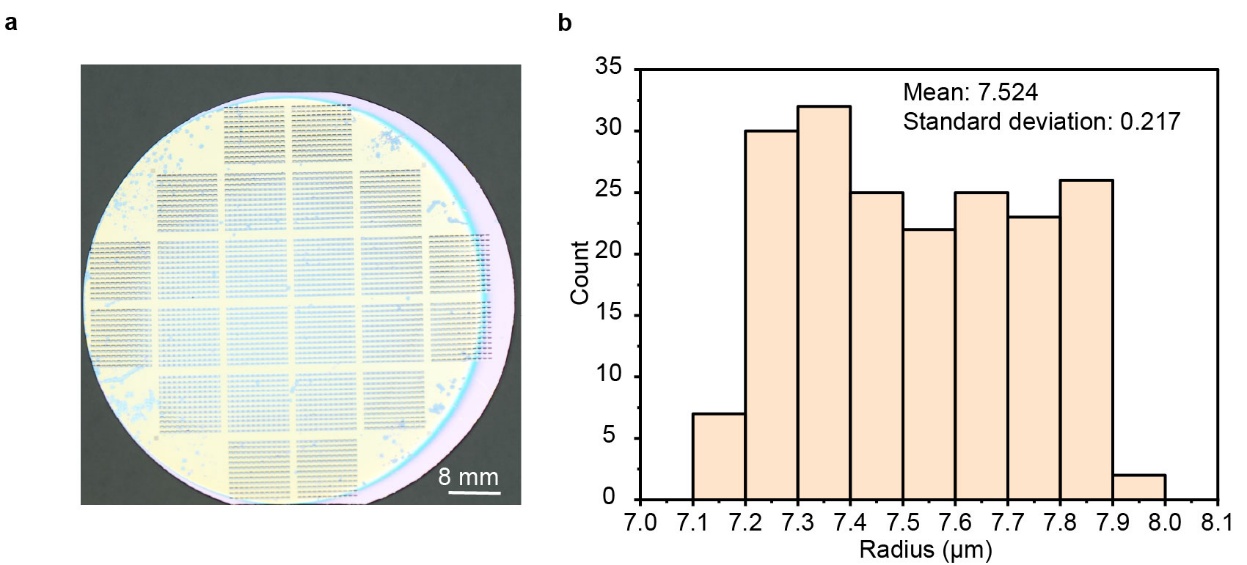


**Figure S3**. The wafer scale fabrication of SiN_x_ microtubes. (a) Optical image of microtube arrays on 2-inch wafer. The microtube arrays consist of 24 sections, with each section comprising a 12 × 12 array. (b) The distribution of microtubes' radius.


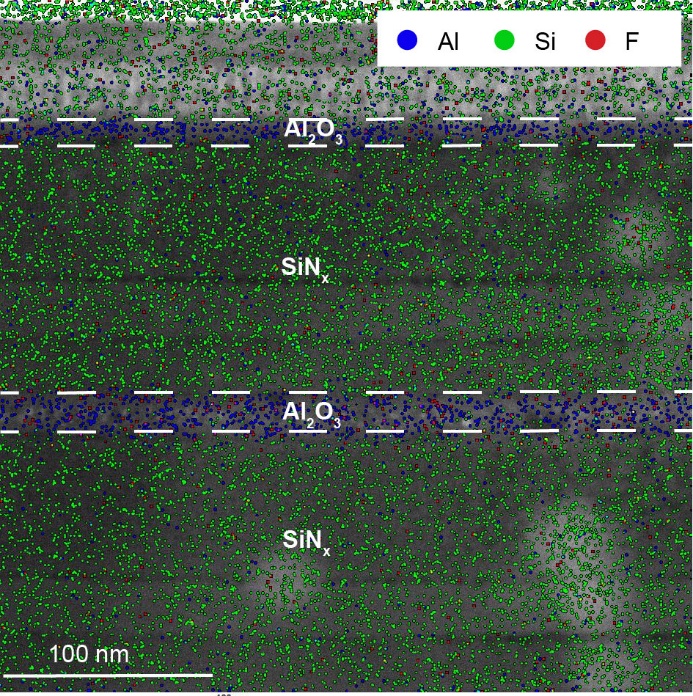


**Figure S4**. The element distribution of Al, Si and F in the cross-section of the wall of microtube characterized by TEM-EDX.


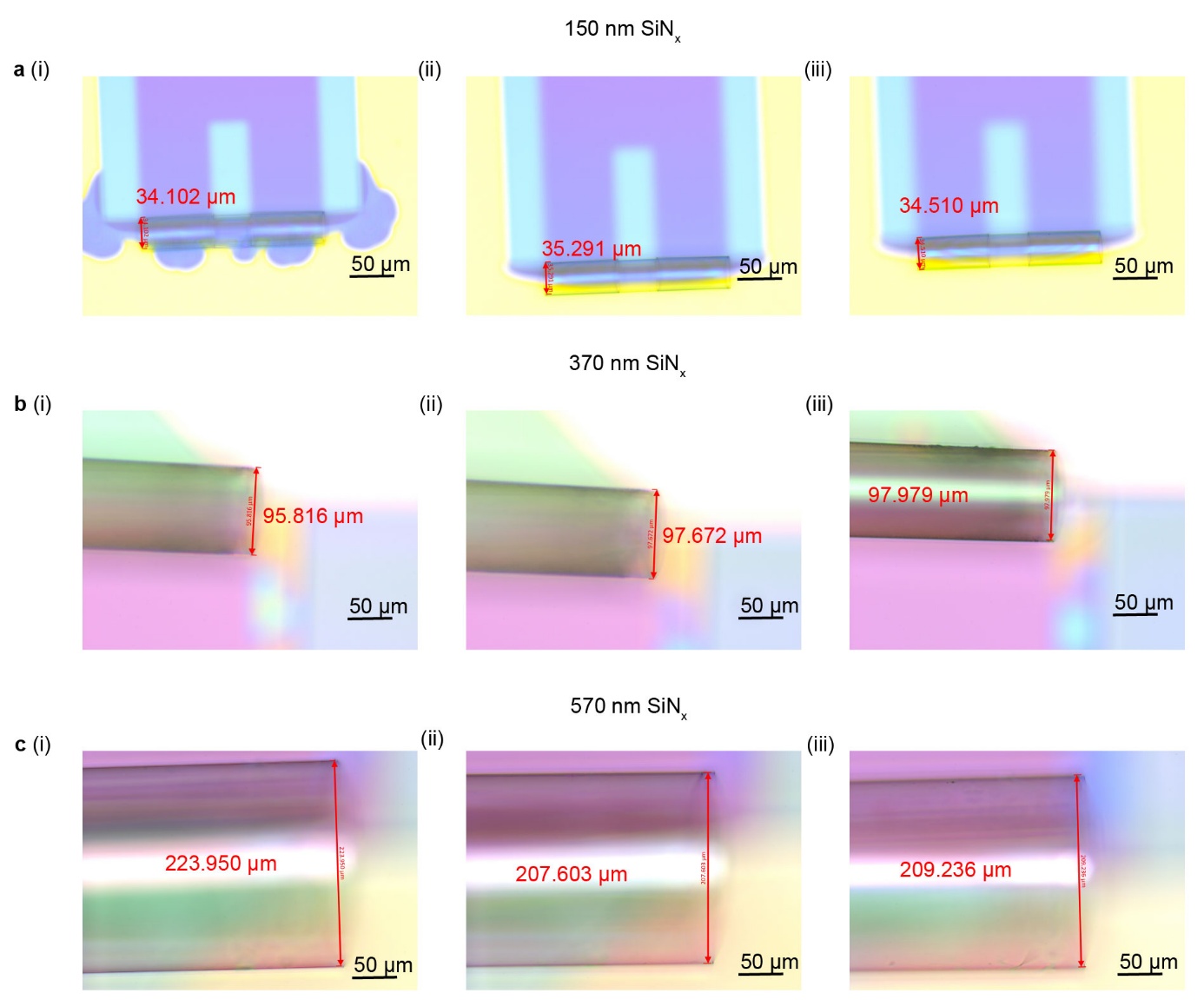


**Figure S5**. Diameter distribution of microtubes. Optical images of microtubes with the thickness of SiN_x_ at **a** 150 nm **b** 370 nm **c** 570 nm. The thickness of SiN_x_ can influence the radius of the self-rolling microtube, and higher thickness of SiN_x_ can lead to higher radius.


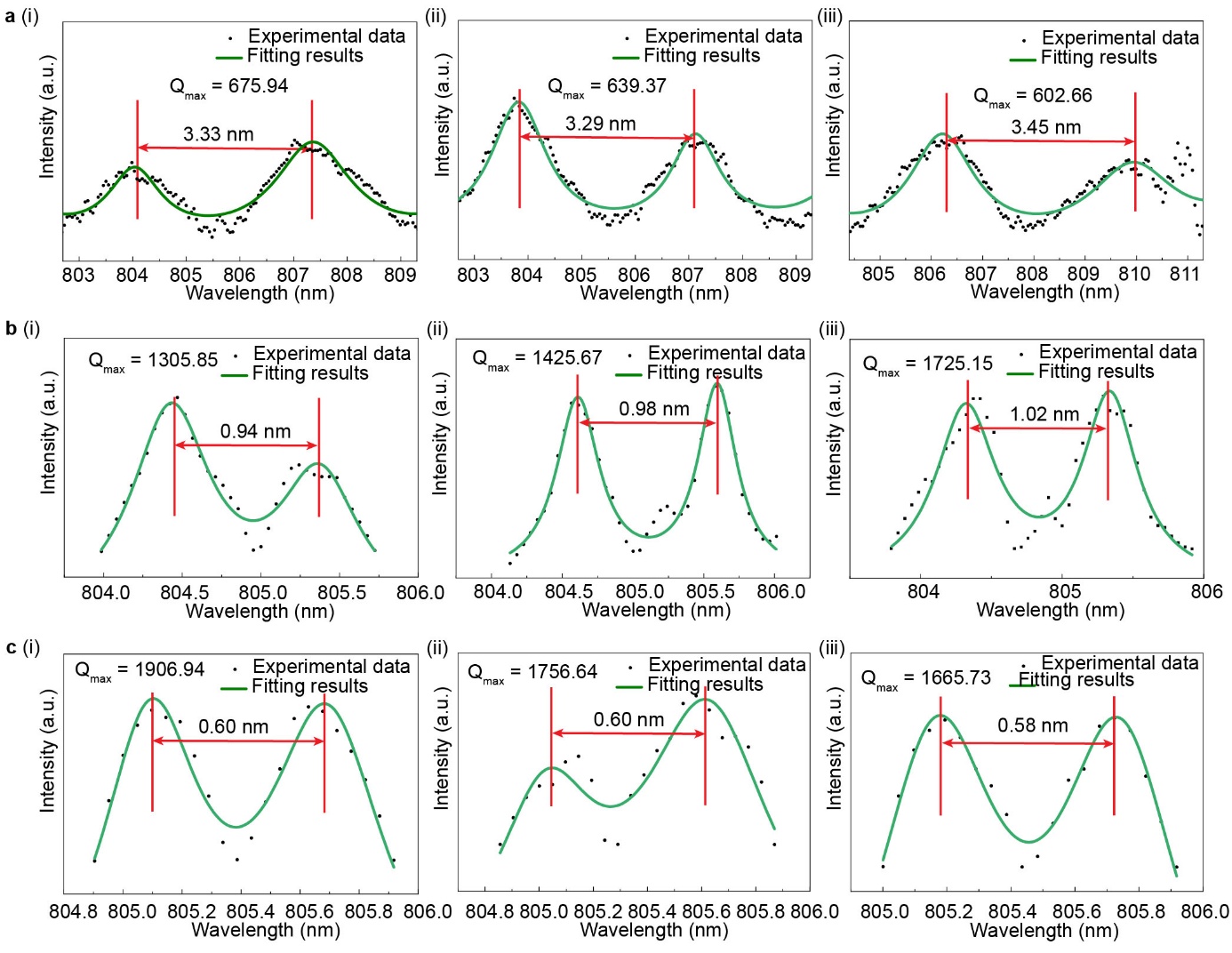


**Figure S6.** PL spectra of microtube resonators in the vicinity of 805 nm. The thickness of SiN_x_ is set at **a** 150 nm **b** 370 nm **c** 570 nm. The peaks in the spectra are fitted with Gaussian curve by the Gaussian deconvolution. As the thickness of SiN_x_ increases, the radii of microtubes increase, leading to the decrease of FSR.


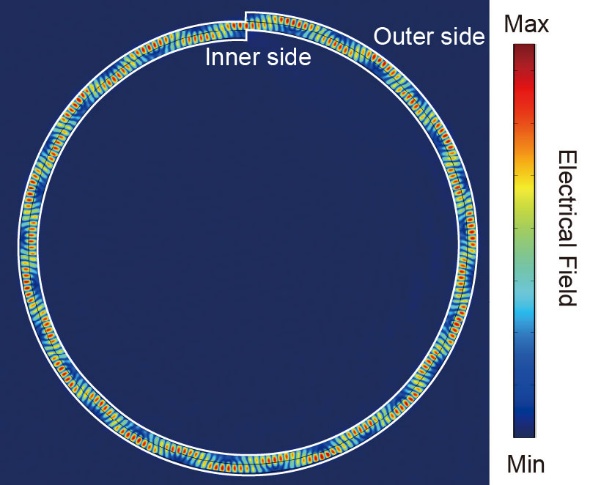


**Figure S7.** The simulated electrical field distribution in the two turn spiral models.


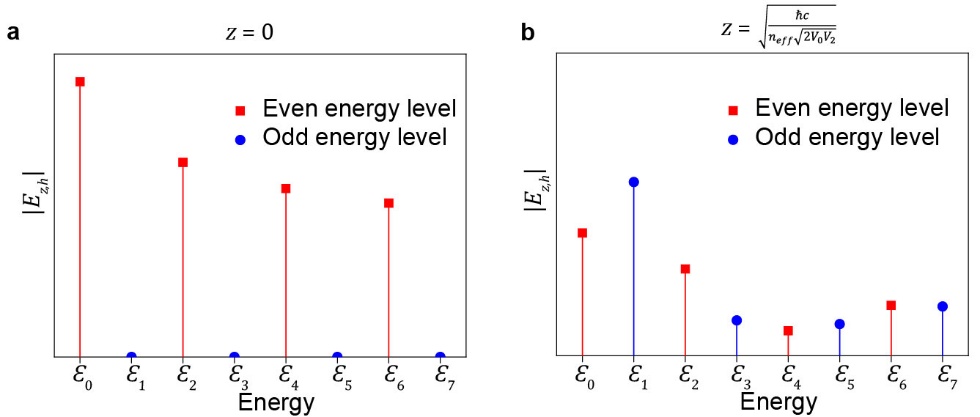


**Figure S8.** Calculated energy levels distributions of microtubes. The energy levels distribution of microtubes is calculated at **a** $z = 0$ and **b** $z =\sqrt{\frac{\hbar c}{n_{eff}\sqrt{2V_{0}V_{2}}}}$. The odd energy levels have no energy distribution at the middle of microtubes ($z = 0$). Near $z = 0$, the energy distribution of odd energy levels will not be 0. Therefore, even though the incident light is focused on the middle of microtubes, the light spot will also overlap with the vicinity of $z = 0$, and the odd energy levels can also be characterized in the PL spectra.


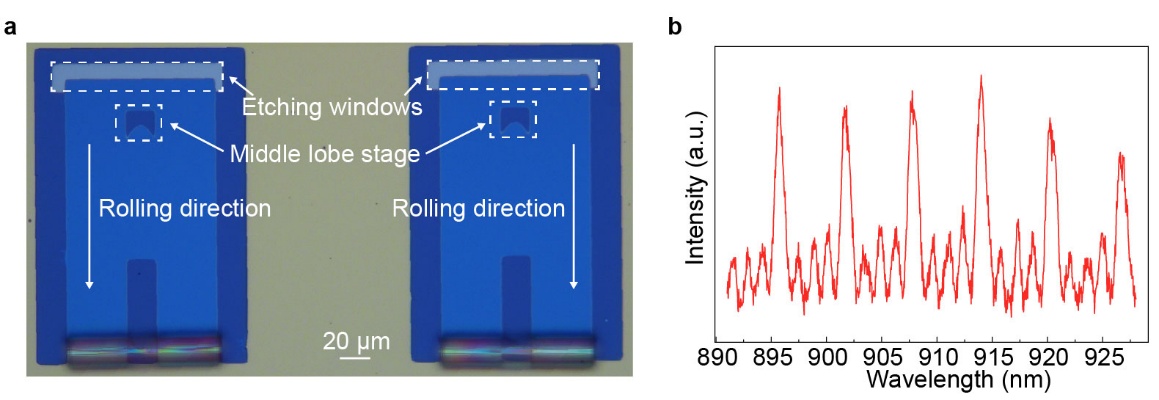


**Figure S9.** The design and testing of self-rolling microtube with lobe structure on the inner side. **a** The self-rolling pattern to realize lobe structure on the inner side of microtube. **b** The PL spectrum of self-rolling microtube with lobe structure on the inner side


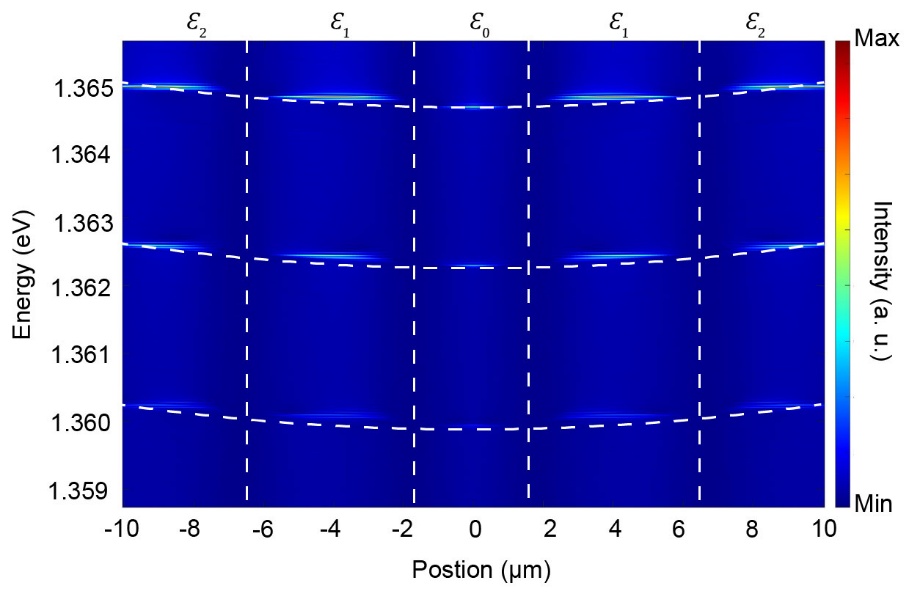


**Figure S10**. The FDTD results of resonating spectra variation along the axial direction.


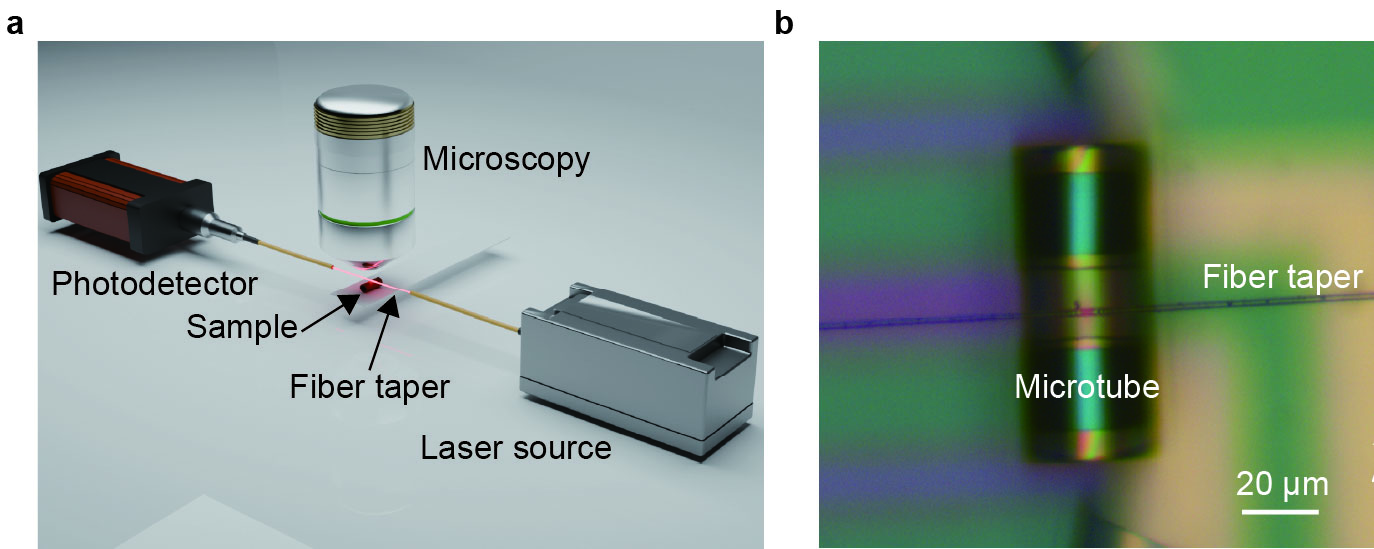


**Figure S11.** Experiment setup for coupling fiber taper with microtube resonator. **a** The schematic diagram of the experimental setup for coupling fiber taper with microtube resonator. **b** The optical microscopy image of the fiber taper contacting with microtube and couples with the resonant cavity in microtube.


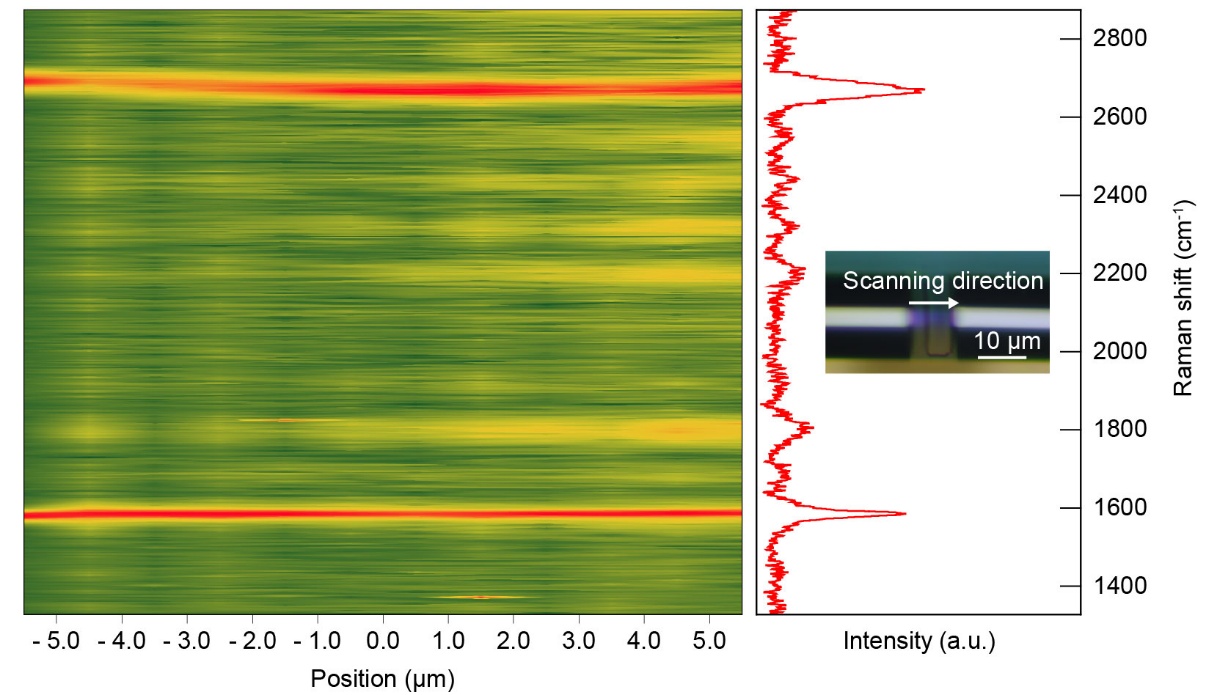


**Figure S12.** Raman line scan along the axis of microtube in the channel area (the middle of microtube is chosen as the position = 0 µm). Left: The Raman spectrum at position = 0 µm. Right: Along the axial path of the microtubule, distinct G characteristic peaks and G’ characteristic peaks can be observed, while the D peak is not obvious. This indicates that Gr is distributed in the channel region with few defects (Inset: the schematical diagram of Raman scanning direction).


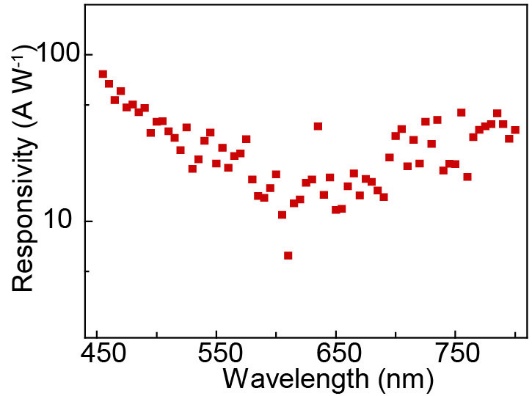


**Figure S13.** The photoresponsivities spectra of Gr-integrated microtube resonators across the visible to near-infrared wavelength range ($\lambda$ = 450-800 nm). The microtube exhibited relative stable photoresponsivities throughout the investigated spectral window.


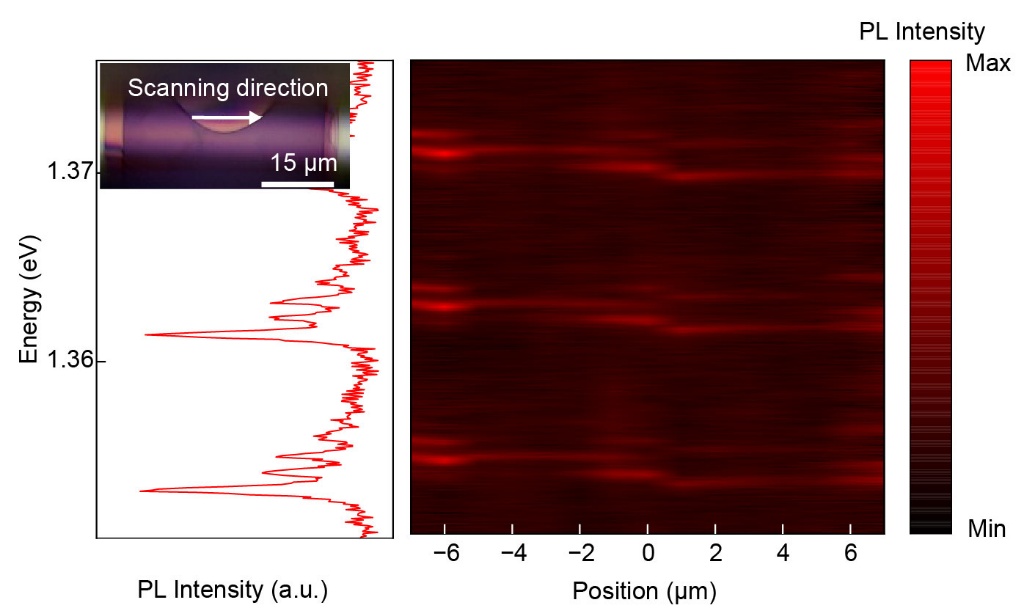


**Figure S14.** PL line scan along the microtube integrated with Gr. Left: PL spectrum of the microtube integrated with Gr at position = 0. (Inset: The schematic diagram of PL scanning direction.) Right: Linear spatial-resolved PL spectrum of the microtube integrated with Gr. The quality factors of resonant peaks and the optical field restriction of lobe structures is weakened after the introduction of Gr.


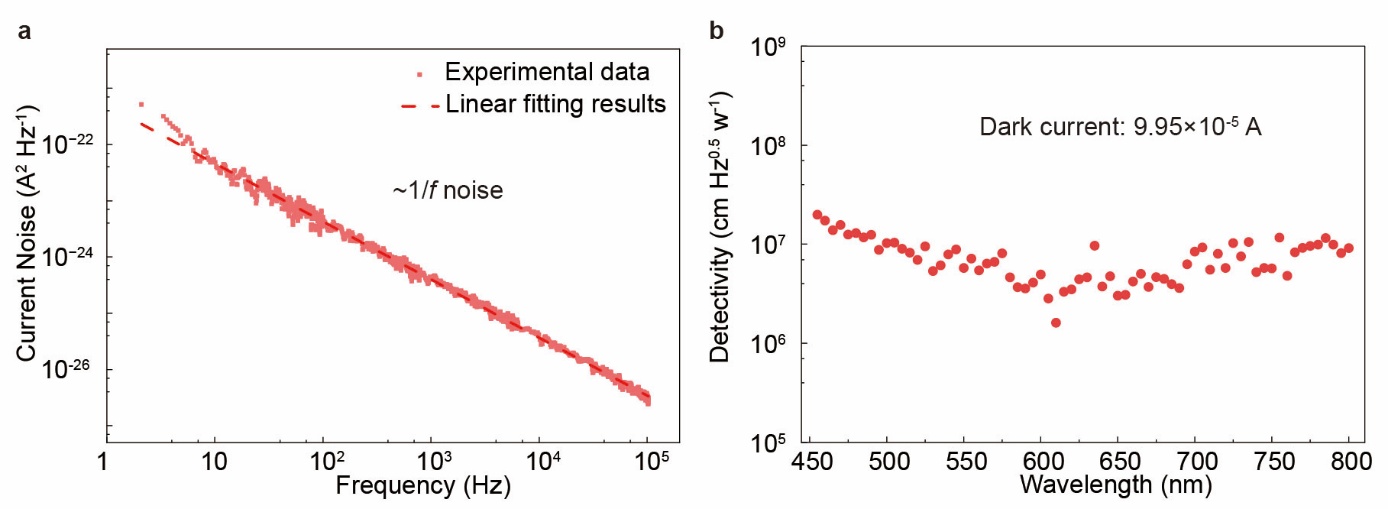


**Figure S15.** The noise characteristics of the graphene integrated microtube. **a** The current noise of the device. **b** the detectivity of the device.


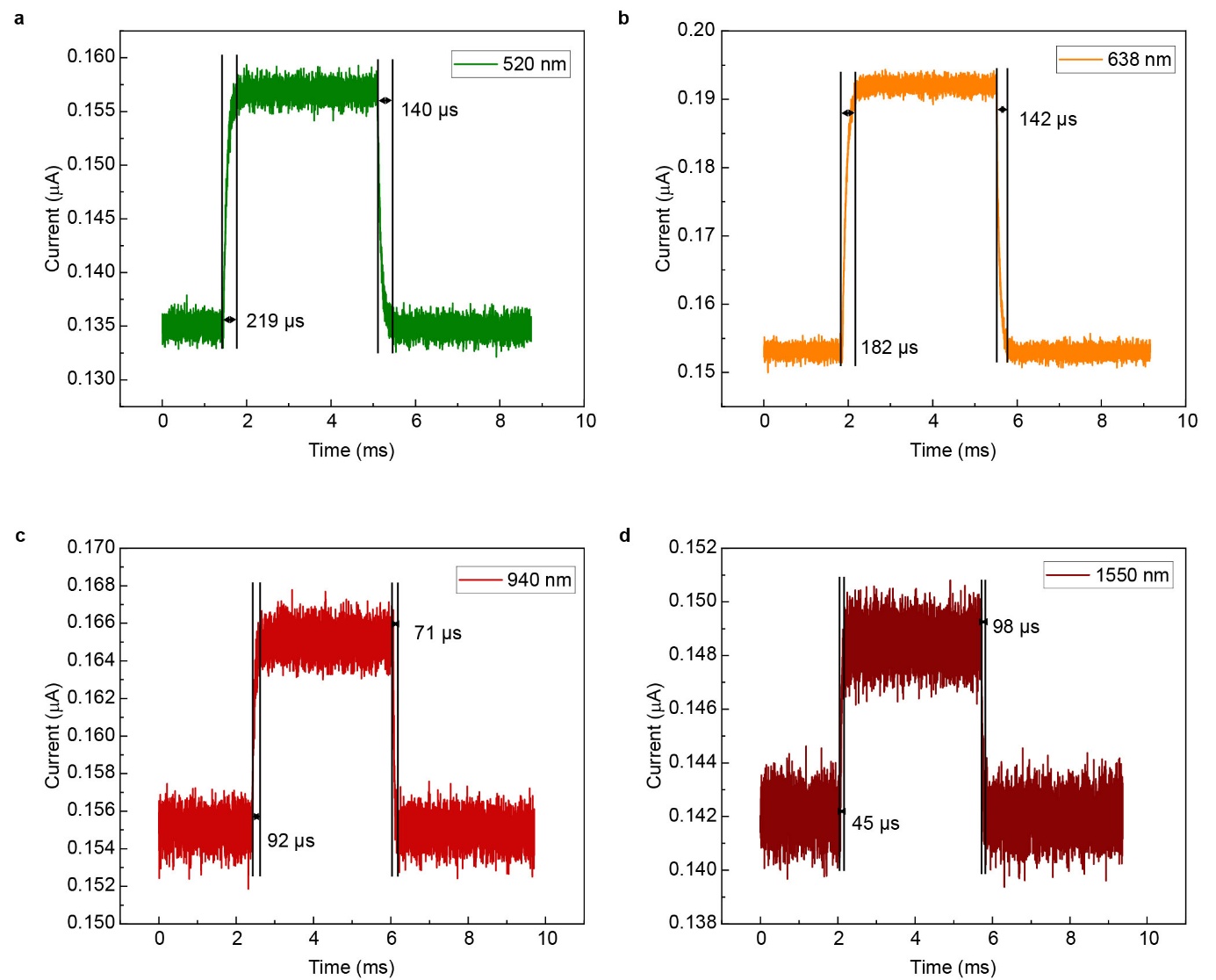


**Figure S16**. The response time of Gr-integrated SiN_x_ microtube at the wavelength of **a** 520 nm **b** 638 nm **c** 940 nm **d** 1550 nm.


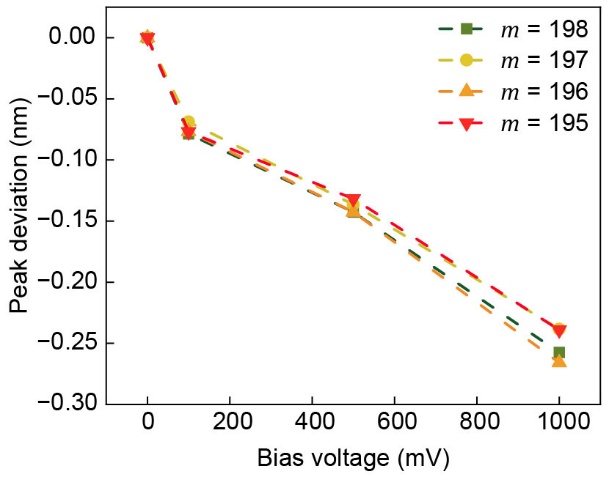


**Figure S17.** Peaks deviation in the PL spectrum of a Gr-integrated microtube resonator under different bias voltage. As the bias voltage increases, the resonant peaks blue shift, due to the decrease of effective refractive index. The peak deviation is calculated by the difference between the wavelength of peaks under bias voltage and the wavelength of peaks under zero bias voltage.


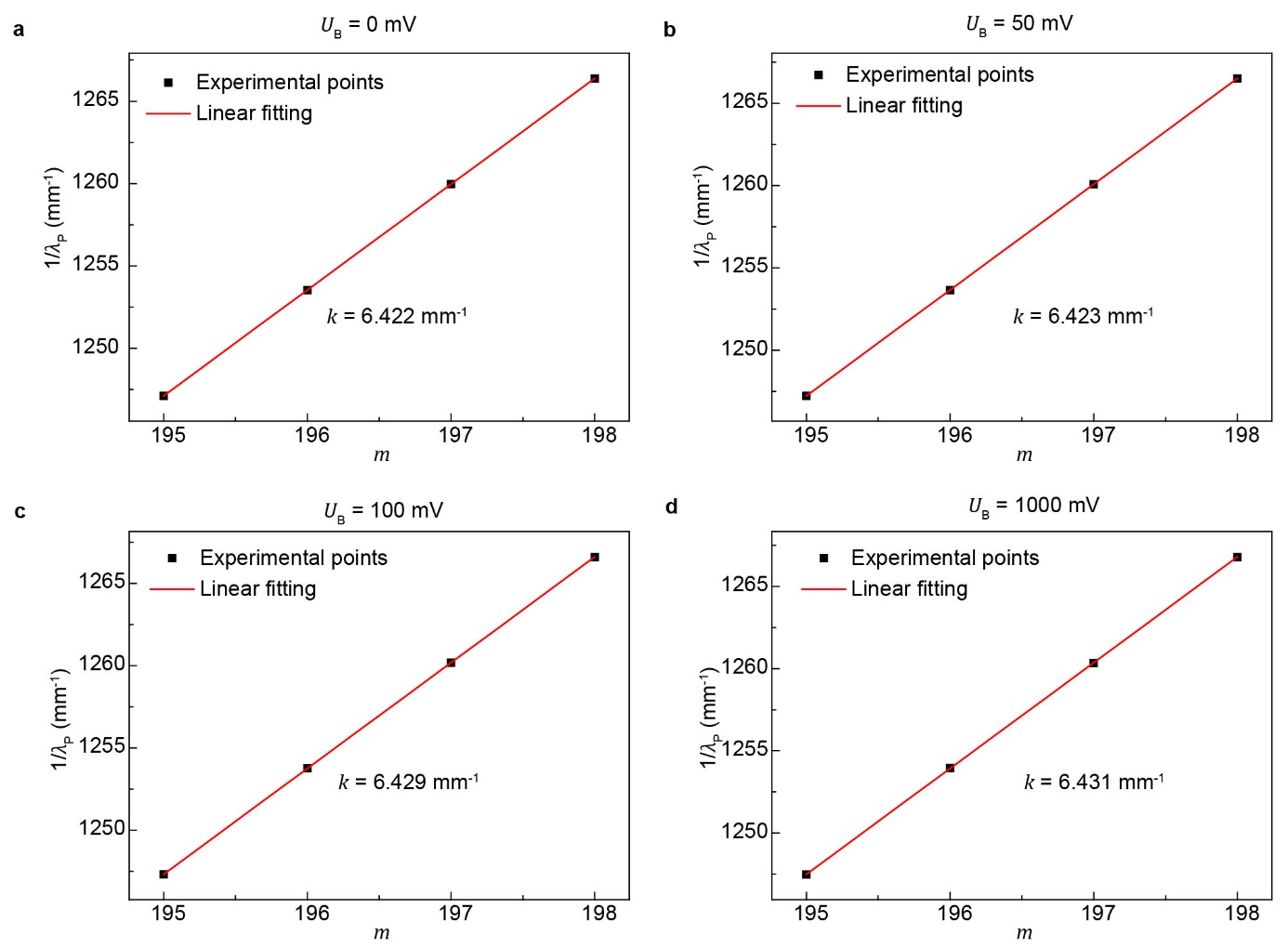


**Figure S18.** Linear fitting of the relationship between $1/\lambda_{P}\lambda_{P}$ and 𝑚 under various bias voltage. The bias voltage is set at **a** 0 mV **b** 50 mV **c** 100 mV **d** 1000 mV. As the bias voltage increases, the slope $k_{\mathrm{slope}}$ of the linear fitting line becomes steeper. Since $k_{\mathrm{slope}}$ is inversely proportional to $n_{\mathrm{eff}}$, the effective refractive index of the resonant cavity decreases with increasing bias voltage.


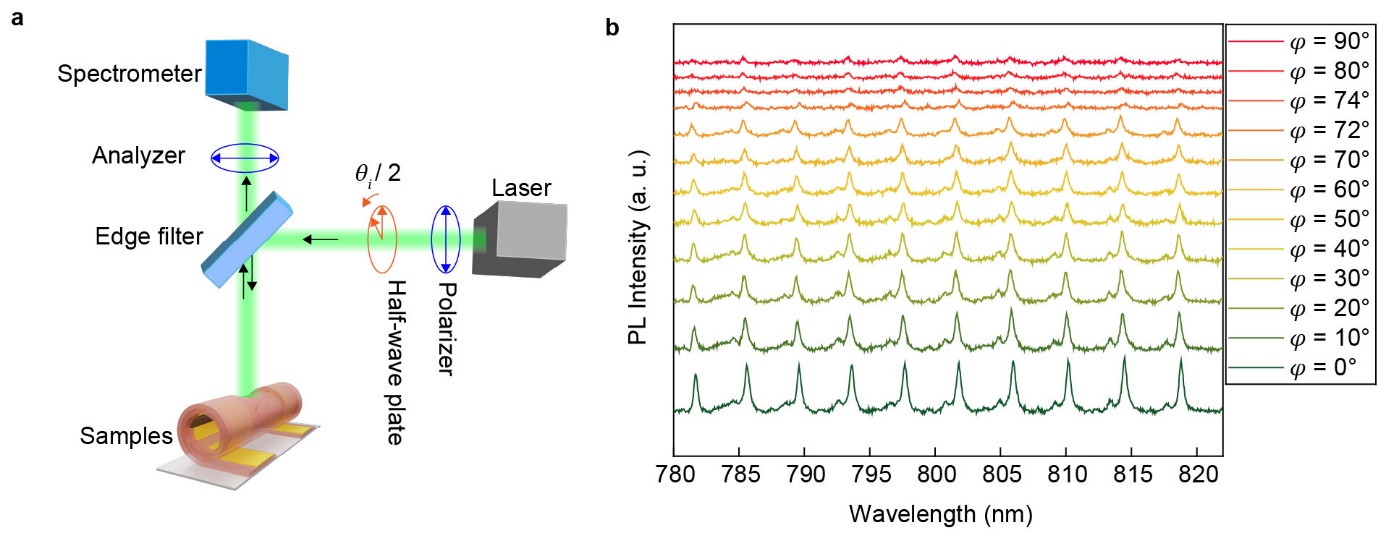


**Figure S19.** PL spectra characterization on the Gr-integrated microtube resonators with different polarization angle. **a** The setup for polarized PL spectra characterization. While maintaining fixed positions of both the scattered light and the sample, the polarization orientation of the incident beam can be systematically manipulated. This manipulation is accomplished by rotating the half-wave plate around its fast axis by an angle of$\theta_{i}/2$, which consequently induces a rotation of $\theta_{i}$ in the polarization direction of the incident light. **b** The PL spectra of the Gr- integrated microtube resonators with the polarization angle $\varphi$ varying from 90° to 0°. As the polarization angle increases, the intensities of resonant peaks decrease. $\varphi=0^{\circ}$ leads to TE mode and the microtube resonators have great selectivity to TE mode, so higher polarization angle means lower TE mode components, resulting in lower resonant peak intensities.


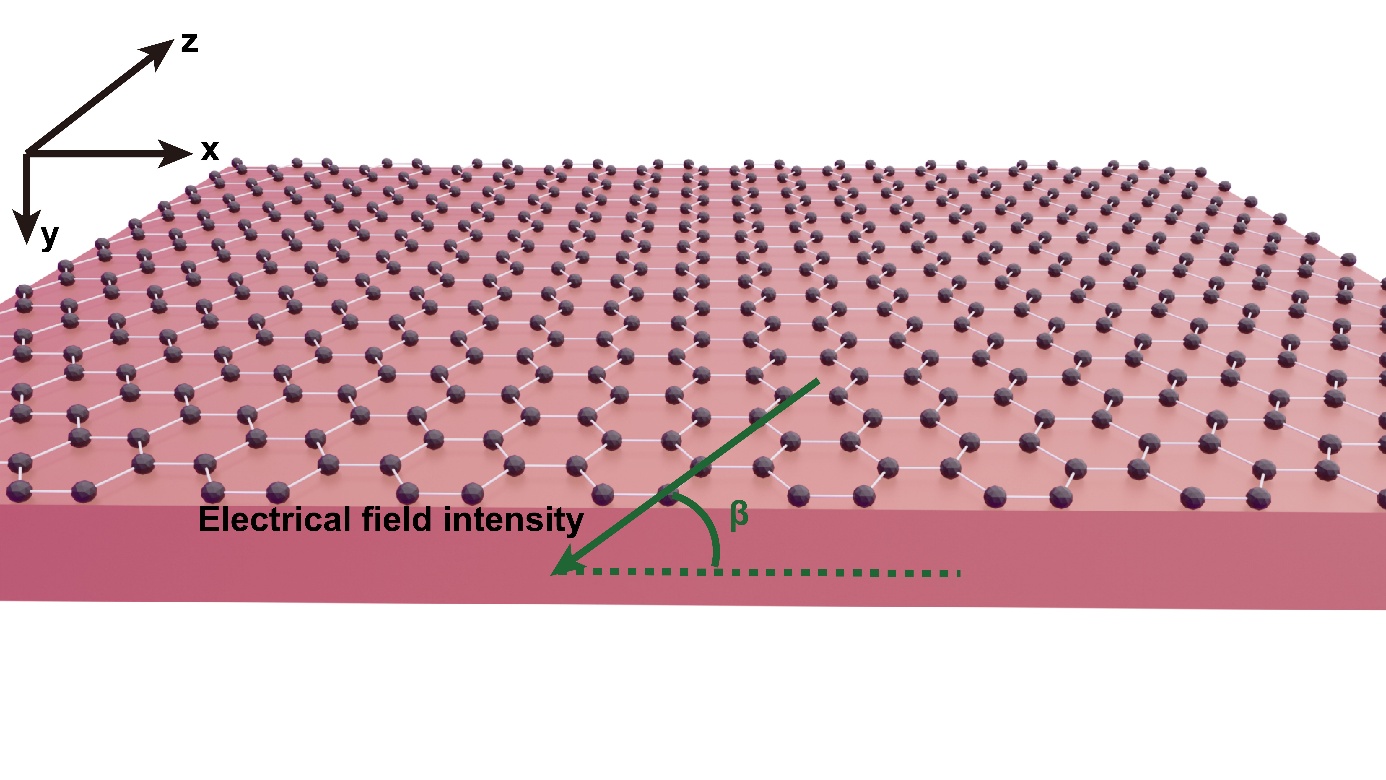


**Figure S20.** The theoretical model for calculating polarization sensitivity of Gr integrated microtubes. $\beta$ is the angle between the electrical field intensity and the plane of Gr. For $\beta=0^{\circ}$, the electrical field intensity is parallel to the plane of Gr, and Gr has high absorptivity to the incident light. For $\beta=90^{\circ}$, the electrical field intensity is perpendicular to the plane of Gr, and Gr almost has no absorption to the incident light.


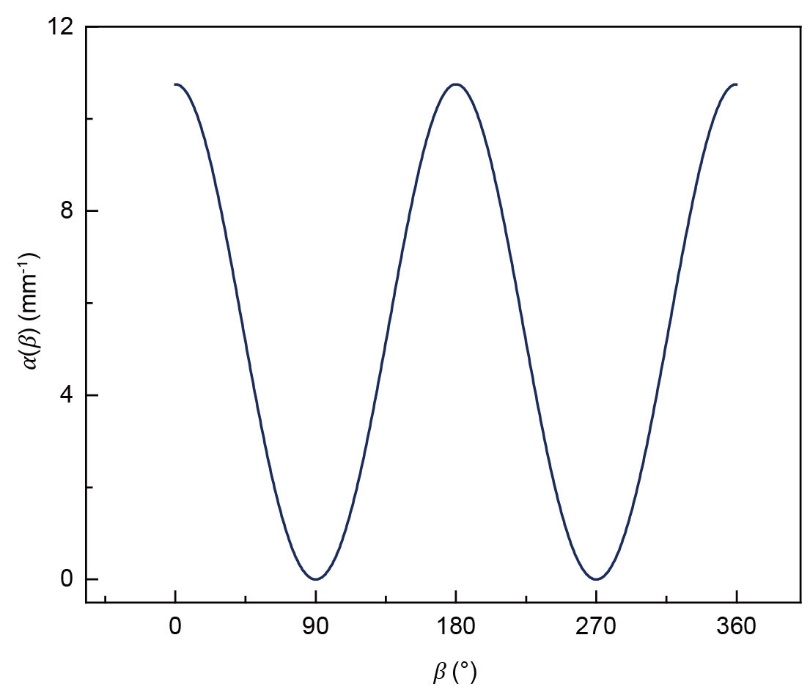


**Figure S21.** Angular dependence of device absorptivity $\alpha\left( \beta\right)$ with incident light wavelength set as 520 nm. $\beta=0^{\circ}$ means that the electrical field intensity is parallel to Gr, so Gr has the highest absorptivity. $\beta=90^{\circ}$ means that the electrical field intensity is perpendicular to Gr, where Gr is theoretically opaque to the incident light.


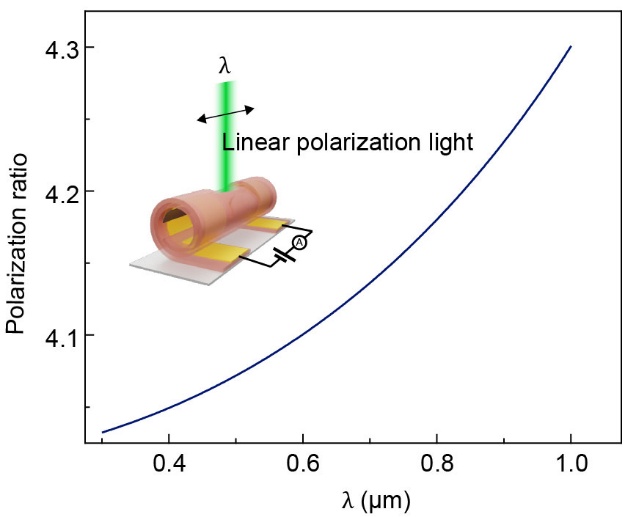


**Figure S22.** Wavelength dependence of device polarization ratio. Within the visible light spectrum, longer wavelengths correspond to higher polarization ratios, arising from the higher absorptivity of Gr at longer wavelengths.


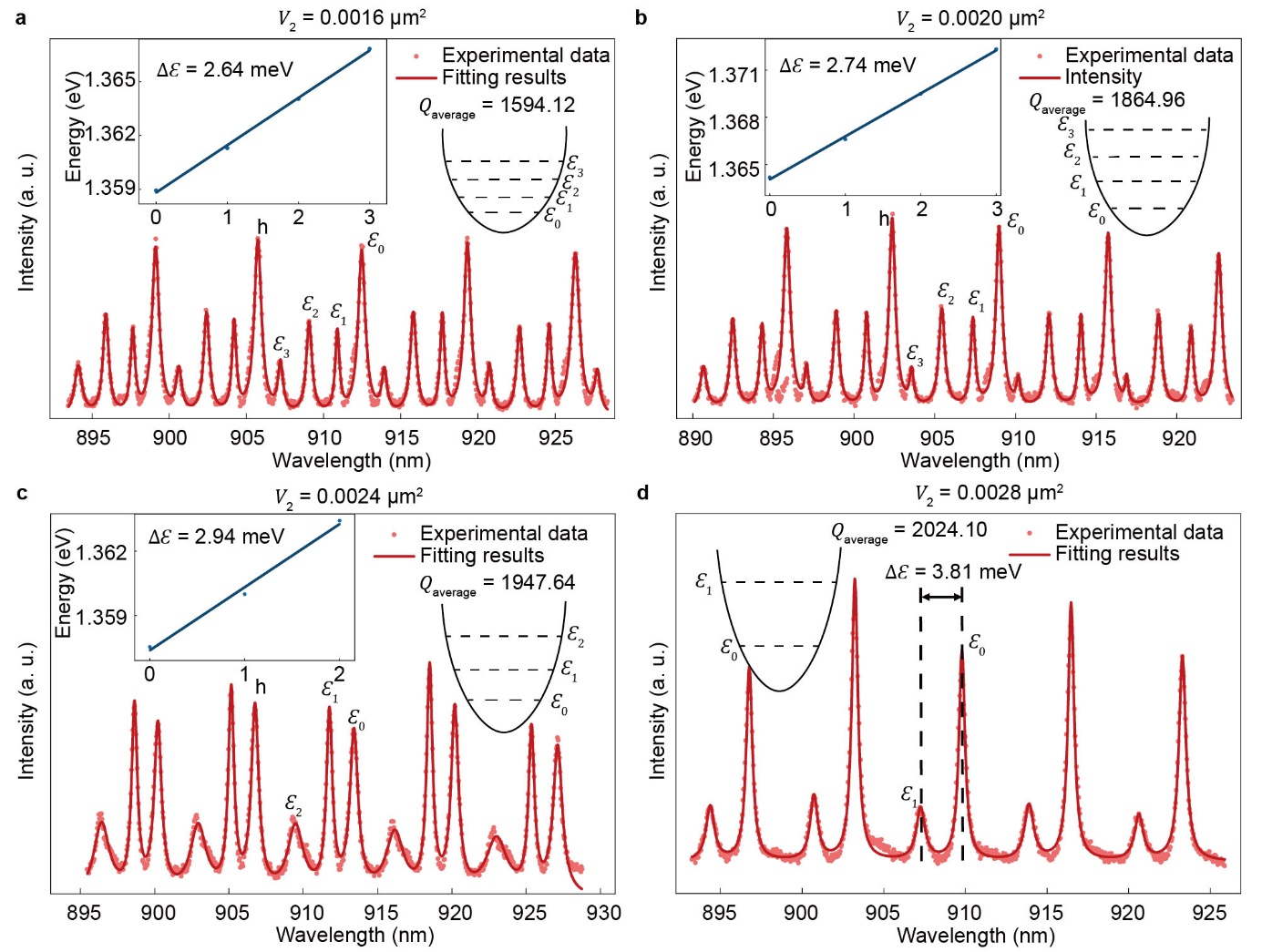


**Figure S23.** The PL spectra of microtube resonators with different parabolic lobe structures. **a** The PL spectra of microtube resonators with $V_{2}=0.0016 \mu m^{-1}$. Inset: the linear fitting results of axial energy levels to calculate the energy level difference. **b** The PL spectra of microtube resonators with $V_{2}=0.0020 \mu m^{-1}$. Inset: the linear fitting results of axial energy levels to calculate the energy level difference. **c** The PL spectra of microtube resonators with $V_{2}=0.0024 \mu m^{-1}$. Inset: the linear fitting results of axial energy levels to calculate the energy level difference. **d** The PL spectra of microtube resonators with $V_{2}=0.0028 \mu m^{-1}$.

**Supplementary Table 1.** Performance comparison between different on-chip optoelectrical devices.

| Main materials | Device | Responsivity | Resonator integration | Material preparation method | Device fabrication method | Footprint | Application | Operating wavelength range | Reference |
| --- | --- | --- | --- | --- | --- | --- | --- | --- | --- |
| Graphene/SiN_x_ | Graphene/ SiN_x_ WGM microtube resonator | 2.8 A W^-1^ | Yes | CVD for graphene/PECVD for SiN_x_ | Wet transfer/lithography/RIE/dry etching | 280 µm^2^ | Optical modulation and detection | 400-1550 nm | This work |
| Graphene/hBN/Si | Graphene/Si WGM microring resonator | 0.5 A W^-1^ | Yes | CVD for graphene/solvent synthesis for hBN/commercial Si waveguide | Wet transfer/EBL/RIE | 5000 µm^2^ | Photodetection | 400-680 nm | 1 |
| Graphene/MoTe_2_/Si | Graphene/MoTe_2_/Si WGM microring resonator | 0.64 mA W^-1^ | Yes | CVD for graphene/CVD for MoTe_2_/commercial SOI | CMP/wet transfer/dry transfer/lithography/RIE | 5000 µm^2^ | Optical modulation and detection | 1530–1565 nm | 2 |
| MoS_2_/SiN_x_ | MoS_2_/SiN_x_ WGM microring resonator | 0.1546 A W^-1^ | Yes | CVD for MoS_2_ and hBN/CVD for SiN_x_ | CMP/dry transfer/lithography/RIE | 70000 µm^2^ | Photodetection | 1500–1630 nm | 3 |
| Graphene oxide/doped silica | Graphene/doped silica WGM microring resonator | N/A | Yes | Solvent synthesis for graphene oxide | CMP/lithography/lift-off | 1100000 µm^2^ | Optical modulation | 1550 nm | 4 |
| AlGaAsOI | AlGaAsOI WGM microring resonator | N/A | Yes | Commercial AlGaAsOI | DUV lithography/dry etching | 70000 µm^2^ | Microcomb | 1530–1570 nm | 5 |
| LNOI | Lithium niobate WGM microring resonator | N/A | Yes | Commercial LNOI | EBL/ICP-RIE | 11000 µm^2^ | Optical parametric oscillator | 1460-1630 nm | 6 |
| Silica | Silica WGM microtoroid resonator | N/A | Yes | Commercial oxidized monocrystalline silicon | 3D printing based on multiphoton lithography | 6000 µm^2^ | Optical modulation | 1530-1610 nm | 7 |
| Silica | Silica WGM microtoroid resonator | N/A | Yes | Commercial oxidized monocrystalline silicon | Lithography/dry etching/laser re-melting technique | 7900 µm^2^ | Chaos-assisted broadband momentum transformation | 500-2500 nm | 8 |
| Er-doped Si | Er-doped Si WGM microdisk resonator | N/A | Yes | Commercial oxidized monocrystalline silicon | Lithography/dry etching | 620 µm^2^ | Microdisk laser | 1550 nm | 9 |
| Lithium niobate | Lithium niobate WGM microdisk resonator | N/A | Yes | Commercial LNOI | EBL/dry etching | 4900 µm^2^ | Optical modulation | 770-1680 nm | 10 |
| Lithium niobate | Lithium niobate WGM microdisk resonator | N/A | Yes | Commercial LNOI | EBL/dry etching | 1300 µm^2^ | Optical modulation | 1540-1550 nm | 11 |
| Ge QD/Si | Ge QD/Si WGM microdisk resonator | 2.13 mA W^-1^ | Yes | MBE for SiGe/Stranski–Krastanov mode for Ge QD/commercial SOI | EBL/dry etching | 50 µm^2^ | Photodetection | 1520-1570 nm | 12 |
| Ge QD/Si | Ge QD/Si WGM microdisk resonator | 5.65 mA W^-1^ | Yes | MBE for SiGe/Stranski–Krastanov mode for Ge QD/commercial SOI | EBL/dry etching | 50 µm^2^ | Light-emitting and photodetection | 1500-1580 nm | 13 |
| MOF/Y_2_O_3_/ZrO_2_ | MOF/Y_2_O_3_/ZrO_2_ WGM microtube resonator | N/A | Yes | ALD and solvent synthesis for MOF/e-beam evaporation for Y_2_O_3_ and ZrO_2_ | Lithography | 2300 µm^2^ | CO_2_ sensing | 630-680 nm | 14 |
| TiO_2_ | TiO_2_ WGM microtube resonator | N/A | Yes | E-beam evaporation for TiO_2_ | Lithography/RIE | 4000 µm^2^ | Optofluidic sensing | 1500-1600 nm | 15 |
| Pd/Y_2_O_3_/ZrO_2_ | Pd/Y_2_O_3_/ZrO_2_ microtube resonator | N/A | Yes | E-beam evaporation for Y_2_O_3_, ZrO_2_ and Pd nanoparticles | Lithography/RIE | 3200 µm^2^ | Gas sensing | 610-680 nm | 16 |
| Graphene/Si/Ge | Graphene/Si_0.4_Ge_0.6_/Si microtube photodetector | 6803 A W^-1^ | No | CVD for graphene/MBE for SiGe and Si | Wet transfer/lithography/wet etching | 540 µm^2^ | Photodetection | 500-1000 nm | 17 |
| Te/SiN_x_ | Te microtube photodetector | 252.13 V W^-1^ | No | Magnetron sputtering for Te/PECVD for SiN_x_ | Lithography/RIE/dry etching | 4000 µm^2^ | Photodetection | 635-1550 nm | 18 |
| Silicon | Silicon microtube photodetector | 330 mA W^-1^ | No | MBE for Si and Ge | Lithography/RIE/wet etching | 840 µm^2^ | Photodetection | 400-1200 nm | 19 |
| Silicon | Silicon-based photonic crystal | N/A | Yes | Commercial SOI | EBL/RIE | 0.28 µm^2^ | Nanocavity | 1560-1580 nm | 20 |
| Graphene/Si | Graphene photodetector/photonic crystal | 0.17 A W^-1^ | Yes | CVD for graphene/Commercial SOI | Wet transfer/EBL/RIE | 82 µm^2^ | Photodetection | 1500-1620 nm | 21 |

Supplementary Notes

**Supplementary Note 1 Derivation of quasi-Schrödinger equation**

Substitute Equation 2 into the Helmholtz equation of electrical field, it can be written as

$$\begin{aligned} \frac{1}{n_{\text{eff}}^{2}}\nabla^{2}\left( E_{\text{c}}E_{z} \right)=k^{2}E_{\text{c}}E_{z}.\#\left( S1 \right) \end{aligned}$$

Because the main dynamics of optical field distribution take place in circular direction due to the strong confinement in radial direction^22^, $\frac{\partial^{2}}{\partial z^{2}}E_{\text{c}}\to0$ and $\nabla^{2}E_{\text{c}}\approx\nabla_{\rho,\theta}^{2}E_{\text{c}}$. Equation S1 can be written as

$$\begin{aligned} \frac{\nabla_{\rho,\theta}^{2}E_{\text{c}}\left( \rho,\theta,z \right)}{E_{\text{c}}\left( \rho,\theta,z \right)}+\frac{1}{E_{z}\left( z \right)}\frac{\partial^{2}}{\partial z^{2}}E_{z}\left( z \right)=-n_{\text{eff}}^{2}k^{2}.\#\left( S2 \right) \end{aligned}$$

For circular optical field distribution, $k_{\text{c}}$ is introduced and defined as

$$\begin{aligned} -n_{\text{eff}}^{2}k_{\text{c}}^{2}=\frac{\nabla_{\rho,\theta}^{2}E_{\text{c}}\left( \rho,\theta,z \right)}{E_{\text{c}}\left( \rho,\theta,z \right)}=\frac{1}{E_{\text{c}}}\left( \frac{\partial^{2}}{\partial\rho^{2}}+\frac{1}{\rho}\frac{\partial}{\partial\rho}+\frac{1}{\rho^{2}}\frac{\partial^{2}}{\partial\theta^{2}} \right)E_{\text{c}}.\#\left( S3 \right) \end{aligned}$$

The optical field is confined in the vicinity of the wall, where $\rho\to R$. Therefore, approximation in $\rho$-direction can be conducted on Equation S3:

$$\begin{aligned} -n_{\text{eff}}^{2}k_{\text{c}}^{2}=\frac{1}{R^{2}E_{\text{c}}}\frac{\partial^{2}}{\partial\theta^{2}}E_{\text{c}}.\#\left( S4 \right) \end{aligned}$$

Considering the periodic boundary condition $E_{\text{c}}\left( R,\theta,z \right)=E_{\text{c}}\left( R,\theta+2\pi,z \right)$, Equation S4 is a typical harmonica equation, and the solution is

$$\begin{aligned} \left\{ \begin{aligned} E_{c,m}=E_{0,m}\left( \rho\right)\exp\left[ ik_{\text{c},m}\left( z \right)n_{\text{eff}}\left( z \right)R\theta\right] \\ k_{\text{c},m}\left( z \right)=\frac{m}{n_{\text{eff}}\left( z \right)R} \end{aligned} \right..\#\left( S5 \right) \end{aligned}$$

For axial optical field distribution,

$$\begin{aligned} \frac{1}{E_{z}\left( z \right)}\frac{\partial^{2}}{\partial z^{2}}E_{z}\left( z \right)-n_{\text{eff}}^{2}k_{\text{c}}^{2}\left( z \right)=-n_{\text{eff}}^{2}k^{2}.\#\left( S6 \right) \end{aligned}$$

By expressing the photon energy $\mathcal{E}$ in terms of $\hbar ck$ and recasting Equation S6 in the form of the Schrödinger equation, we obtain the quasi-Schrödinger equation:

$$\begin{aligned} \hat{H}_{\text{q}}E_{z}\left( z \right)\mathcal{=E}E_{z}\left( z \right).\#\left( S7 \right) \end{aligned}$$

**Supplementary Note 2 The eigenvalues and eigenfunctions of quasi-Schrödinger equation with parabolic shape quasi-potential**

In this work, we designed the lobe structure with a parabolic profile, resulting in a parabolic distribution of self-rolling turns along the microtube axis. Given the positive correlation between self-rolling turns and $n_{\text{eff}}$,^23^ combined with the relation $V_{\text{q}}\left( z \right)=\hbar c\frac{m}{n_{\text{eff}}\left( z \right)R}$, the quasi-potential distribution function can be approximated as a parabolic function:

$$\begin{aligned} V_{\text{q}}\left( z \right)=V_{0}+V_{2}z^{2},\#\left( S8 \right) \end{aligned}$$

where $V_{0}$ is the quasi-potential at $z=0$, corresponding to the vertex of the parabola, while $V_{2}$ represents its quadratic coefficient. Considering that $n_{\text{eff}}$ changes slowly along the axis of microtube, the quartic coefficient of $V_{\text{q}}^{2}$ can be neglected. Substitute Equation S8 into the quasi-Schrödinger equation:

$$\begin{aligned} \left[ -\left( \frac{\hbar c}{n_{\text{eff}}} \right)^{2}\frac{\partial^{2}}{\partial z^{2}}+V_{0}^{2}+2V_{0}V_{2}z^{2} \right]E_{z}\left( z \right)=\mathcal{E}^{2}E_{z}\left( z \right).\#\left( S9 \right) \end{aligned}$$

Equation S9 is a typical quantum harmonic oscillator equation, and its eigenfunctions are the Hermite functions:

$$\begin{aligned} \left\{ \begin{aligned} \mathcal{E}_{h}=\sqrt{V_{0}^{2}+\frac{\hbar c}{n_{\text{eff}}}\left( 2h+1 \right)\sqrt{2V_{0}V_{2}}} \\ E_{\text{z},h}=\sqrt{\frac{1}{2^{h}h!}\frac{n_{\text{eff}}}{\hbar c}}\left( \frac{2V_{0}V_{2}}{\pi} \right)^{\frac{1}{4}}\exp\left( -\frac{n_{\text{eff}}}{\hbar c}\sqrt{\frac{V_{0}V_{2}}{2}}z^{2} \right)H_{h}\left( \sqrt{\frac{n_{\text{eff}}}{\hbar c}\sqrt{2V_{0}V_{2}}}z \right) \end{aligned}, \right.h\in N^{*},\#\left( S10 \right) \end{aligned}$$

where $H_{h}$ are the $h$-order Hermite polynomials. Given that the resonant mode changes subtly along the axis of microtube, which means $\frac{\hbar c}{n_{\text{eff}}}\left( 2h+1 \right)\sqrt{2V_{0}V_{2}}\ll V_{0}^{2}$, $\mathcal{E}_{h}$ can be rewritten into an equidistant form by introducing Taylor approximation:

$$\begin{aligned} \mathcal{E}_{h}\approx V_{0}+\left( h+\frac{1}{2} \right)\frac{\hbar c}{n_{\text{eff}}}\sqrt{\frac{2V_{2}}{V_{0}}}.\#\left( S11 \right) \end{aligned}$$

This equation shows that the axial energy level difference $\Delta\mathcal{E=}\frac{\hbar c}{n_{\text{eff}}}\sqrt{\frac{2V_{2}}{V_{0}}}$ depends on the quasi-potential shape determined by lobe structure. We therefore investigated geometric optimization to enhance Q factor while maintaining photodetection efficiency. We fabricated parabolic lobe structures with $V_{2}$ parameters of 0.0016, 0.0020, 0.0024, and 0.0028 $\mu m^{-1}$. The photoluminescence spectra in Figure S16 show clear correlation between structural curvature and optical performance. As $V_{2}$ increases from 0.0016 to 0.0028 $\mu m^{-1}$, the axial energy level spacing enlarges and Q factor improves from 1594.12 to 2024.10. This Q factor enhancement results from improved optical confinement through geometric optimization. Linear fitting in Figure S16 reveals that increased parabolic curvature (higher $V_{2}$) enlarges energy level differences, suppressing axial photon propagation losses by making inter-level transitions more energetically demanding. The enhanced energy separation confines photons within their axial modes, reducing propagation losses and improving Q factor. Higher curvature creates steeper potential gradients that better prevent axial photon leakage while preserving radial field distribution and light-graphene interaction strength for photodetection efficiency.

**Supplementary Note 4 The energy losses during light propagation**

Considering the energy losses during the light propagation, the complex refractive index $\tilde{n}=n_{\mathrm{eff}}(1+i\kappa)$ can be introduced into Equation S11:

$$\begin{aligned} {\tilde{\mathcal{E}}}_{h}=V_{0}+\left( h+\frac{1}{2} \right)\frac{\hbar c}{n_{\mathrm{eff}}\left( 1+i\kappa\right)}\sqrt{\frac{2V_{2}}{V_{0}}},\#\left( S12 \right) \end{aligned}$$

where $\kappa$ is the effective extinction coefficient. Therefore, the wavelength of light can be calculated:

$$\begin{aligned} \tilde{\lambda}_{h}=\frac{2\pi\hbar c}{{\tilde{\mathcal{E}}}_{h}}=\frac{2\pi\hbar c}{V_{0}}\left[ 1-\left( h+\frac{1}{2} \right)\frac{\sqrt{2V_{0}V_{2}}}{V_{0}^{2}}\frac{\hbar c}{n\left( 1+\kappa^{2} \right)}\left( 1-i\kappa\right) \right].\#\left( S13 \right) \end{aligned}$$

Then we can calculate the Q factor:

$$\begin{aligned} Q_{h}=\left| \frac{Re\left( \tilde{\lambda}_{h} \right)}{Im\left( \tilde{\lambda}_{h} \right)} \right|=\frac{1}{\kappa}-\frac{V_{0}^{2}n\left( 1+\kappa^{2} \right)\kappa}{\left( h+\frac{1}{2} \right)\sqrt{2V_{0}V_{2}}\hbar c}\approx\frac{1}{\kappa}.\#\left( S14 \right) \end{aligned}$$

The effective extinction coefficient can be decomposed:

$$\begin{aligned} \kappa=\kappa_{\mathrm{in}}+\kappa_{\mathrm{mat}}=\kappa_{\mathrm{in}}+\frac{t_{\mathrm{Gr}}l_{\mathrm{Gr}}}{t_{\mathrm{tot}}l_{\mathrm{tot}}}\kappa_{\mathrm{Gr}},\#\left( S15 \right) \end{aligned}$$

where $\kappa_{\mathrm{in}}$ is the intrinsic extinction comes from the structure of microtube resonators and $\kappa_{\mathrm{mat}}$ comes from the light absorption of materials. $t_{\mathrm{Gr}}$ and $t_{\mathrm{tot}}$ are the thickness of graphene and the perimeter of microtubes, respectively. $l_{\mathrm{Gr}}$ and $l_{\mathrm{tot}}$ are the thickness of graphene and the wall of microtubes, respectively. Considering that the absorption of SiN_x_ is much smaller than that of graphene, we can only consider the extinction coefficient of graphene $\kappa_{\mathrm{Gr}}$:

$$\begin{aligned} \kappa_{\mathrm{Gr}}=\frac{t_{\mathrm{tot}}l_{\mathrm{tot}}}{t_{\mathrm{Gr}}l_{\mathrm{Gr}}}\left( \kappa-\kappa_{\mathrm{in}} \right)=\frac{t_{\mathrm{tot}}l_{\mathrm{tot}}}{t_{\mathrm{Gr}}l_{\mathrm{Gr}}}\left( \frac{1}{Q}-\frac{1}{Q_{0}} \right),\#\left( Re5 \right) \end{aligned}$$

where $Q_{0}$ is the Q factor of the microtube without graphene. The calculated extinction of the different length ($L=10,20,30,40 \mu m$) graphene mentioned in Figure 4e are 0.21, 0.11, 0.12 and 0.36, respectively, which are consistent with the extinction coefficient of graphene reported previously^24^.

**Supplementary Note 4 The angular dependence of device absorptivity**

Stemming from the anisotropic nature of Gr 2D crystalline structure, the relative permittivity tensor of the Gr/SiN_x_ heterostructure assumes the tensor form in the principal coordinate system:

$$\begin{aligned} \tilde{\varepsilon}=\left[ \begin{matrix} \tilde{\varepsilon}_{x} & 0 & 0 \\ 0 & \tilde{\varepsilon}_{y} & 0 \\ 0 & 0 & \tilde{\varepsilon}_{z} \end{matrix} \right],\#\left( S16 \right) \end{aligned}$$

where $\tilde{\varepsilon}_{x}$, $\tilde{\varepsilon}_{y}$ and $\tilde{\varepsilon}_{z}$ are the relative permittivity of the nanomembrane along the direction of $\hat{x}$, $\hat{y}$ and $\hat{z}$ respectively (Figure S13), which are defined by:^17^

$$\begin{aligned} \left\{ \begin{matrix} \tilde{\varepsilon}_{x}=\tilde{\varepsilon}_{z}=\tilde{\varepsilon}_{\text{Si}\text{N}_{\text{x}}}-A\left( 1-i\frac{2\Gamma}{\omega} \right) \\ \tilde{\varepsilon}_{y}=\tilde{\varepsilon}_{\text{Si}\text{N}_{\text{x}}} \\ \begin{matrix} \tilde{\varepsilon}_{\text{Si}\text{N}_{\text{x}}}=\varepsilon_{\text{Si}\text{N}_{\text{x}}}+i\frac{\sigma_{\text{Si}\text{N}_{\text{x}}}}{\omega} \\ A=\frac{\mu_{\text{c}}e^{2}}{\pi\hbar^{2}\left( \omega^{2}+4\Gamma^{2} \right)\varepsilon_{0}\Delta}>0 \end{matrix} \end{matrix} \right.,\#\left( S17 \right) \end{aligned}$$

where $\varepsilon_{\text{Si}\text{N}_{\text{x}}}$ and $\sigma_{\text{Si}\text{N}_{\text{x}}}$ refer to the relative permittivity and conductivity of SiN_x_ respectively, $\mu_{\text{c}}$ is the chemical potential of the material, e is the electron charge, $\omega$ is the angular frequency of the photon, $\hbar$ is the reduced Planck constant, $\Gamma$ is the scattering rate and $\Delta$ is the thickness of the nanomembrane. The relative permittivity of SiN_x_ is dependent on the wavelength of incident light.^25^

The electric field intensity is written as:

$$\begin{aligned} \tilde{\boldsymbol{E}}\left( \beta\right)=\left[ \begin{matrix} \cos\beta\\ \sin\beta\\ 0 \end{matrix} \right]\tilde{E},\#\left( S18 \right) \end{aligned}$$

where $\beta$ is the angle between $\tilde{\boldsymbol{E}}$ and the surface of Gr, as illustrated in Figure S13. Therefore, the relative permittivity along the direction of $\tilde{\boldsymbol{E}}$ can be solved by:

$$\begin{aligned} \left\{ \begin{aligned} \tilde{\varepsilon}\left( \beta\right)=\frac{{\tilde{\boldsymbol{E}}}^{T}\vec{\vec{\varepsilon}}\tilde{\boldsymbol{E}}}{\tilde{E}^{2}}=\varepsilon_{\text{Si}\text{N}_{\text{x}}}+i\frac{\sigma_{\text{Si}\text{N}_{\text{x}}}}{\omega}-A\left( 1-i\frac{2\Gamma}{\omega} \right)\cos^{2} \beta\\ \varepsilon\left( \beta\right)=Re\left( \tilde{\varepsilon} \right)=\varepsilon_{\text{Si}\text{N}_{\text{x}}}-A\cos^{2} \beta\\ \sigma\left( \beta\right)=\omega Im\left( \tilde{\varepsilon} \right)=\sigma_{\text{Si}\text{N}_{\text{x}}}+2A\Gamma\cos^{2} \beta\end{aligned} \right..\#\left( S19 \right) \end{aligned}$$

Therefore, absorptivity can be solved by:

$$\begin{aligned} \alpha\left( \beta\right)=\frac{2\pi c}{\lambda}\sqrt{\sqrt{\varepsilon^{2}\left( \beta\right)+\left( \frac{\lambda\sigma\left( \beta\right)}{2\pi c} \right)^{2}}-\varepsilon\left( \beta\right)}.\#\left( S20 \right) \end{aligned}$$

**Supplementary Note 5 Calculation of the polarization ratio**

As for polarization parallel to the axis of the tubular, the electrical field intensity is parallel to the surface of Gr ($\beta=0$), and the absorbance coefficient of the incident light can be written as:

$$\begin{aligned} \alpha_{\parallel}=\alpha\left( 0 \right)=\frac{2\pi c}{\lambda}\sqrt{2\mu\left[ \sqrt{\varepsilon^{2}\left( 0 \right)+\left( \frac{\sigma\left( 0 \right)}{\omega} \right)^{2}}-\varepsilon\left( 0 \right) \right]}.\#\left( S21 \right) \end{aligned}$$

The average absorbance of the incident light can be written as:

$$\begin{aligned} \left\{ \begin{aligned} \text{ratio}\left( \lambda\right)=\alpha\left( 0 \right)\frac{\int_{-\frac{l}{2}}^{\frac{l}{2}} \int_{0}^{\frac{\pi}{2}} I_{\text{in}}\left( \rho,\beta,z \right)d\beta dz}{\int_{-\frac{l}{2}}^{\frac{l}{2}} \int_{0}^{\frac{\pi}{2}} \alpha\left( \beta\right)I_{\text{in}}\left( \rho,\beta,z \right)d\beta dz} \\ I_{\text{in}}\left( \rho,\beta,z \right)=\left\{ \begin{aligned} 1&, \left( \rho\cos\beta-R \right)^{2}+z^{2}\leq r_{\mathrm{in}} \\ 0&, \left( \rho\cos\beta-R \right)^{2}+z^{2}>r_{\mathrm{in}} \end{aligned} \right. \end{aligned} \right.,\#\left( S22 \right) \end{aligned}$$

where $I_{\text{in}}\left( \rho,\beta,z \right)$ represents the spatial intensity distribution of the incident light, $r_{\text{in}}$ is its spot radius, and $l$ is the effective length of the photoresponse channel. Therefore, the ratio of $\alpha_{\parallel}$ and $\left. <\alpha_{\perp} \right.>$ can be written as:

$$\begin{aligned} \text{ratio}\left( \lambda\right)=\frac{\alpha_{\parallel}}{\left. <\alpha_{\perp} \right.>}=\alpha\left( 0 \right)\frac{\int_{-\frac{l}{2}}^{\frac{l}{2}} \int_{0}^{\frac{\pi}{2}} \int_{0}^{R} I_{\text{in}}\left( \rho,\beta,z \right)d\rho d\beta dz}{\int_{-\frac{l}{2}}^{\frac{l}{2}} \int_{0}^{\frac{\pi}{2}} \int_{0}^{R} \alpha\left( \beta\right)I_{\text{in}}\left( \rho,\beta,z \right)d\rho d\beta dz}.\#\left( S23 \right) \end{aligned}$$

**Supplementary References**

1. Schuler, S. et al. High-responsivity graphene photodetectors integrated on silicon microring resonators. Nature Communications **12**, 3733 (2021).
2. Wu, J. et al. Dual-function optical modulation and detection in microring resonators integrated graphene/MoTe_2_ heterojunction. Applied Physics Reviews **11**, 021426 (2024).
3. Zhang, Q. et al. High-responsivity MoS_2_ hot-electron telecom-band photodetector integrated with microring resonator. Applied Physics Letters **120**, 261111 (2022).
4. Zhang, Y. et al. Design and Optimization of Four-Wave Mixing in Microring Resonators Integrated With 2D Graphene Oxide Films. Journal of Lightwave Technology **39**, 6553-6562 (2021).
5. Bai, B. et al. Microcomb-based integrated photonic processing unit. Nature Communications **14**, 66 (2023).
6. Lu, J. et al. Ultralow-threshold thin-film lithium niobate optical parametric oscillator. Optica **8**, 539 (2021).
7. Gao, H. et al. 3D printed on-chip microtoroid resonators and nested spiral photonic devices. Photonics Research **9**, 1803 (2021).
8. Jiang, X. et al. Chaos-assisted broadband momentum transformation in optical microresonators. Science **358**, 344-347 (2017).
9. Kippenberg, T. J., Kalkman, J., Polman, A., Vahala, K. J. Demonstration of an erbium-doped microdisk laser on a silicon chip. Physical Review A **74**, 051802 (2006).
10. Wang, C. et al. Integrated high quality factor lithium niobate microdisk resonators. Optics Express **22**, 30924 (2014).
11. Wang, J. et al. High-Q lithium niobate microdisk resonators on a chip for efficient electro-optic modulation. Optics Express **23**, 23072 (2015).
12. Wang, T.-J., He, J.-Y., Lee, C.-A., Niu, H. High-quality LiNbO_3_ microdisk resonators by undercut etching and surface tension reshaping. Optics Express **20**, 28119 (2012).
13. Xu, X., Chiba, T., Maruizumi, T., Shiraki, Y. Microdisk enhanced photodetector based on Ge self-assembled quantum dots on silicon-on-insulator. Thin Solid Films **557**, 363-367 (2014).
14. Kong, Y. et al. Integration of a Metal-organic framework film with a tubular whispering-gallery-mode microcavity for effective CO_2_ sensing. ACS Applied Materials & Interfaces **13**, 58104-58113 (2021).
15. Madani, A. et al. Optical microtube cavities monolithically integrated on photonic chips for optofluidic sensing. Optics Letters **42**, 486-489 (2017).
16. Yang, S. et al. Enhanced evanescent field coupling of smart particles in tubular optical microcavity for sensing application. Advanced Optical Materials **10**, 2102158 (2022).
17. Zhang, Z. et al. Graphene readout silicon-based microtube photodetectors for encrypted visible light communication. Advanced Materials **37**, e2413771 (2025).
18. Huang, J. et al. Enhanced photothermoelectric conversion in self-rolled tellurium photodetector with geometry-induced energy localization. Light: Science & Applications **13**, 153 (2024).
19. Wu, B. et al. Self-rolled-up ultrathin single-crystalline silicon nanomembranes for on-chip tubular polarization photodetectors. Advanced Materials **35**, e2306715 (2023).
20. Akahane, Y., Asano, T., Song, BS. et al. High-Q photonic nanocavity in a two-dimensional photonic crystal. Nature **425**, 944–947 (2003).
21. Schuler, S., Schall, D., Neumaier, D., et al. Graphene photodetector integrated on a photonic crystal defect waveguide. ACS Photonics **5**, 4758-4763 (2018).
22. Strelow, C. et al. Light confinement and mode splitting in rolled-up semiconductor microtube bottle resonators. Physical Review B **85**, 155329 (2012).
23. Strelow, C. et al. Three dimensionally confined optical modes in quantum-well microtube ring resonators. Physical Review B **76**, 045303 (2007).
24. Tikuišis, K., Dubroka, A., Uhlířová, K., et al. Dielectric function of epitaxial quasi-freestanding monolayer graphene on Si-face 6H-SiC in a broad spectral range. Physical Review Materials **7**, 044201 (2023).
25. Beliaev, L. Y., Shkondin, E., Lavrinenko, A. V., Takayama, O. Optical, structural and composition properties of silicon nitride films deposited by reactive radio-frequency sputtering, low pressure and plasma-enhanced chemical vapor deposition. Thin Solid Films **763**, 139568 (2022).
